# Recurrent circadian fasting (RCF) improves blood pressure, biomarkers of cardiometabolic risk and regulates inflammation in men

**DOI:** 10.1186/s12967-019-2007-z

**Published:** 2019-08-19

**Authors:** Iftikhar Alam, Rahmat Gul, Joni Chong, Crystal Tze Ying Tan, Hui Xian Chin, Glenn Wong, Radhouene Doggui, Anis Larbi

**Affiliations:** 1 Department of Community Health Sciences, Clinical Nutrition Program, College of Applied Medical Sciences, King Saud University, King Abdullah Street, Riyadh, Kingdom of Saudi Arabia; 2 Department of Human Nutrition and Dietetics, Bacha Khan University, Charsaddah, KPK Pakistan; 3 Biology of Aging Laboratory, Singapore Immunology Network, Agency for Science Technology and Research, 8A Biomedical Grove, Singapore, 138648 Singapore; 4 SURVEN (Nutrition Surveillance and Epidemiology in Tunisia) Research Laboratory, National Institute of Nutrition and Food Technology (INNTA), Tunis, Tunisia; 5 Department of Microbiology and Immunology, Yong Loo Lin School of Medicine, National University of Singapore, Singapore, Singapore

**Keywords:** Recurrent fasting, Inflammation, Health benefits, Aging

## Abstract

**Background:**

The effects of fasting on health in non-human models have been widely publicised for a long time and emerging evidence support the idea that these effects can be applicable to human practice.

**Methods:**

In an open label longitudinal follow-up, a cohort of 78 adult men (aged 20 to 85 years) who fasted for 29 consecutive days from sunrise to sunset (16 h fasting—referred to as recurrent circadian fasting) in Pakistan, were studied. The primary outcomes of the fasting study was weight loss/recovery and the associated changes in blood pressure and circulating levels of surrogate markers linked to organ and system functions—including cardiovascular, metabolic and inflammation. Post-fasting outcomes include the regulation of physiological biomarkers.

**Results:**

Recurrent circadian fasting with weight loss reduced blood pressure (140.6 vs. 124.2 mmHg) and markers of cardiovascular risk (~ 4-fold for resistin; triglycerides: p < 0.0001). Reduced glycemia (p < 0.0001) and the associated changes in the regulation of ketosis (β-hydroxybutyrate) were accompanied by a metabolic shift (PPARβ, osteoprotegerin), suggesting the involvement of the different physiological systems tested. Elevated orexin-A levels (p = 0.0183) in participants indicate sleep disturbance and circadian adaptation. All participants had CRP level < 2 mg/l during the fasting period and a similar trend was observed for TNFα. While most SASP molecules were decreased after the fasting period, heightened levels of IL-8 and IL-6 suggest that some inflammatory markers may be elevated by recurrent circadian fasting. Importantly, older adults reveal similar or more substantial benefits from fasting.

**Conclusions:**

Recurrent circadian fasting is beneficial at the cardiometabolic and inflammatory levels, especially for at-risk individuals—this is contingent on compliance towards the recommended dietary behaviour, which controls carbohydrate and caloric intake. These benefits from fasting may be particularly beneficial to older adults as they often exhibit abnormal cardiovascular, metabolic and inflammatory signatures.

**Electronic supplementary material:**

The online version of this article (10.1186/s12967-019-2007-z) contains supplementary material, which is available to authorized users.

## Background

In order to translate the immunological benefits of caloric restriction from animal models into human studies, practical strategies must be devised to facilitate compliance. The effects of fasting, particularly intermittent fasting (IF), have revealed promising results in recent years and have been shown to be linked to metabolic regulation and occasional intentional weight loss [1]. Intermittent fasting is linked to improvement in the levels of biomarkers associated with cardiovascular health (blood pressure), diabetes (insulin and glucose levels), oxidative stress (8-isoprostane) and inflammation (IL-6, CRP)—suggestive of a systemic impact. Other dietary adjustments, in the form of fasting mimicking diets or time-restricted feeding (TRF), have also contributed to moderate weight loss and significant cardiometabolic health improvements [2].

While different forms of IF are being thoroughly investigated to identify an optimal practice, the health impact of traditional forms of fasting have been neglected despite their widespread practice [3]. For example, fasting during Ramadan—a form of recurrent fasting (i.e. the practice of fasting for periods of 15 days or more)—is a tradition in Asia, Africa and the Middle East. This fasting—which occurs between sunrise and sunset—lasting 29/30 consecutive days, stimulates weight loss and synchronizes with our circadian rhythm: we refer this practice as recurrent circadian fasting (RCF). RCF is a unique model of fasting and credits scientific attention as the scarcity of adverse health reports related to its practice, in spite of its historical persistence and universal participation, identifies it as a safe and sustainable form of fasting. High-risk groups whose constitution may suffer from include pregnant women, individuals suffering from metabolic disorders (e.g. diabetes) and the elderly, as the latter are prone to suffer from malnutrition, physical frailty, and age-associated co-morbidities [4]. The most distinctive aspects of RCF, as compared to other forms of fasting, relates to its continuity (lack of interval between fasting days) and duration.

While some studies suggest that TRF may have few beneficial effects [5], these were referenced against intermittent fasting and may be compounded by non-compliance [3, 6], and diverse eating habits [7]. A recent study by Jakubowicz et al. [8] has revealed the benefits of consuming a high-calorie breakfast, as opposed to forgoing breakfast, in modulating “*clock*” gene expression, postprandial glycemia and body weight [8]. Furthermore, combining a high-caloric breakfast with a low-caloric supper suppresses glycemia in type-2 diabetes and results in better management of metabolic syndrome in overweight women. Altogether, these studies suggest potential health benefits in the practice of RCF, which allows only early and late caloric consumption [9]. Nevertheless, a main concern with prolonged fasting protocols, is non-compliance or heterogeneity in eating behaviour which affects our ability to interpret the potential health benefits of fasting [10].

We now know that fasting results in ketogenesis, promotes potent changes in metabolic pathways and cellular processes such as stress resistance, lipolysis and autophagy, and can have medical applications that in some cases can become an alternative strategy to medication [11, 12]. RCF may alter the circadian consumption and distribution of energy and other nutrients for the fulfilment of daily requirements [13, 14]. A recent meta-analysis suggested that breakfast may not be recommended for individuals whose intention is to lose weight [15]. On a related note, recent studies have demonstrated a negative impact of forgoing breakfast [16, 17], and late night eating habits on cardiometabolic health [18]. Fasting, in general, has been reported to improve several risk factors for stroke and coronary artery diseases, including a reduction in blood pressure and increased insulin sensitivity [19–21]. However, taking into account the restricted time of dietary and nocturnal eating during RCF, it is imperative to understand the effects of dietary alterations on various physiologic systems, and to estimate their possible contribution to the overall health and quality of life outcomes of RCF practitioners. The effects of disrupted sleep and feeding patterns during RCF on a broad range of inflammatory and other biological biomarkers have not been studied before. Therefore, in this pilot study, we examined the changes in the diurnal expression of selected cytokines, metabolic profile and anthropometric indices in healthy Pakistani adults at baseline, at the end and after RCF is completed. In addition, we investigated possible associations with selected novel and traditional cardiometabolic risk factors.

## Methods

### Study design and population

**Fig. 1:**
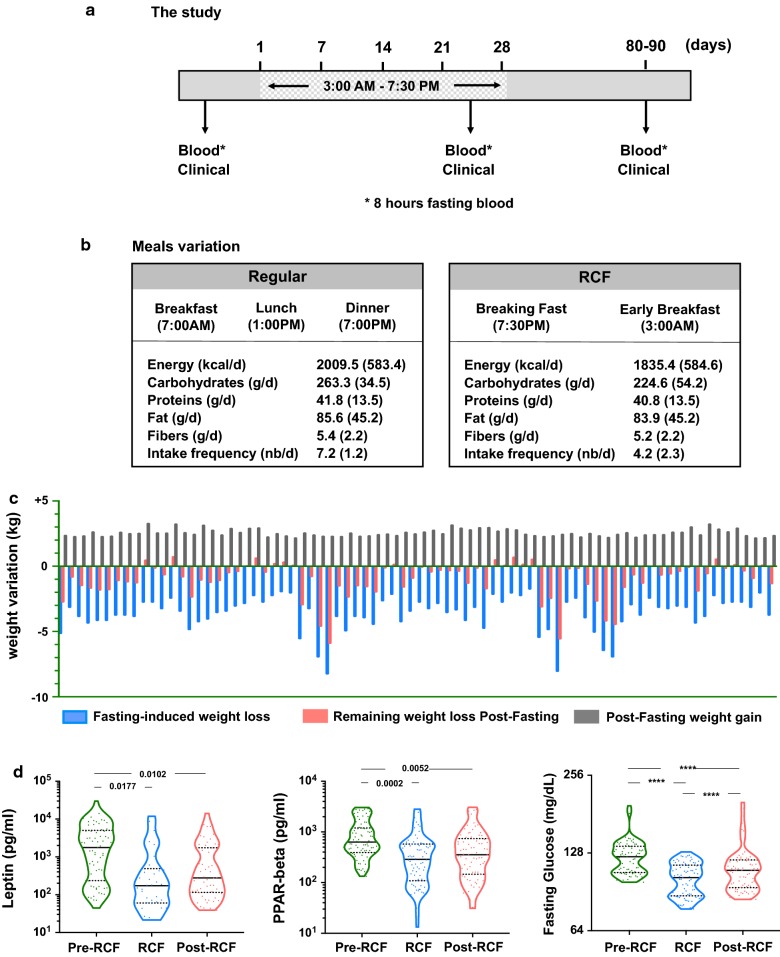
Study design and meal variations. **a** Study design and **b** main variations in meal timing and intake. **c** Change in absolute weight (kg) in the study population during and after fasting. **d** Metabolic adaptation to fasting assessed by leptin, PPAR and fasting glucose levels

The study employed an open label longitudinal follow-up design with convenience sampling to obtain three measurement points: baseline (before fasting), end of fasting (4 weeks) and post-fasting (1 month) (Fig. 1a). The target population was adults aged 20 years and above, belonging to eight Union Councils within Peshawar, Charsadda (central), Malakand and Swat (northern) districts of Khyber Pakhtunkhwa (KPK), Pakistan. The subjects were contacted through NEAT (nutrition, education, awareness and training), a health organization registered with the Government of Pakistan, Department of Social Welfare, KPK, Pakistan. NEAT advocates nutrition awareness and counselling in KPK, Pakistan. Addresses and mobile/phone numbers of participants were obtained from the address directory of NEAT. A SMS was sent to subjects who fulfilled the inclusion/exclusion criteria to invite them to participate in this study. A 1-day workshop was arranged for potential participants to educate them about the study design and purpose. Written informed consents were obtained from the participants during the workshop. The inclusion criteria were to include otherwise medically healthy subjects with no recent past history of infectious or uncontrolled non-communicable chronic diseases. The study was approved by the Research Ethics and Review Board of NEAT (Social Welfare Department, Govt. of Pakistan). While both genders were targeted for the study, only few females (n = 5) participated which did not allow sufficient numbers for inclusion in the analysis. By tradition, females rarely contribute to such studies in presence of males. In 2016, Ramadan fasting began on the 6th of June and ended on the 4th of July, lasting for 29 days. Humidity (40%) and temperatures was consistent throughout the duration and averaged 38/30 °C, with the minimum and maximum at 35/29 °C and 41/38 °C respectively. The average fasting duration was 16 h, lasting between dawn and sunset. The baseline survey was conducted 1 week before the start of fasting and an ongoing survey was conducted during the last week (week 4) of fasting. The final survey (post-fasting survey) was conducted 1 month after the end of fasting (1st week of August).

### Power calculation and data collection

We calculated sample size based on weight loss, our primary outcome, and the necessary physiological adaptation when RCF is conducted appropriately. In average, a loss of 0.8 kg per week is achieved during RCF based on our observation from previous RCF analysis. This open label longitudinal follow-up study, was powered to detect a mean change of 0.1 kg/day between the fasting and post-fasting periods. With α = 0.05 and β = 0.8, we required n = 34 participants to detect a change of 3.0 kg for the whole RCF period. As we were also interested in older adults [22], we therefore aimed at recruiting a final sample of n > 34 individuals < 35 years and n > 34 individuals > 50 years old. The final number of participants to this study was n = 100 and we could collect samples and analyse the respective blood from n = 78 adult males, including n = 37 young and n = 41 older adults.

Data were obtained through pre-tested, semi-structured questionnaires. One questionnaire elicited information related to the socio-demographic, fasting and dietary behaviour of the participant. The same set of questionnaires was used for all follow-up surveys. Subjects were also instructed to communicate the consumption of any special food items during the fasting period. Data collection was performed by student members of NEAT who received special training on questionnaire administration and anthropometric measurements before each assessment. Field supervisors were also deployed to oversee and perform on-site questionnaire checks to ensure completeness and same-day rectification of incomplete responses and measurements. Electronic weighing scales were used for all weight measurements (± 0.1 kg). Body mass index (BMI) was calculated as weight/height^2^ (kg/m^2^), and body fat was measured using the Bio-impedance Analysis (BIA) technique (Bodystat 1500, Bodystat, Isle of Man, UK).

### Dietary data

Dietary data were collected using semi-quantitative Food Frequency Questionnaire (FFQ) by conducting face-to-face interviews as well as the 24 h dietary recall. In these sessions, subjects were asked to recall their nutritional intake—including breakfast and their final meal of the day. Information on the cooking method (fried, boiled, steamed, roasted) and source of food (home-cooked vs. outsourced) were also collected. During the interview, an adult of the household would be present to corroborate the nutritional report so as to ensure accurate verbal reporting. Serving size was quantified in terms of cups, bowls, and spoons using a specified reference (for instance, half of the small bowl). When interviewees gave unspecific answers (e.g. ‘*I used a lot of milk in my tea’*), they were prompted to convert their estimation to cup equivalents. The average food intake over these 3 days was calculated and used to estimate consumption. Nutritional content was computed based on food composition tables specific for the Pakistani context and extensively used in our previous studies [23, 24]; the analysed categories included total energy, fat, protein, carbohydrate and selected vitamins and minerals. The percentage of energy derived from fat, protein, and carbohydrate were also calculated. Given the distinction in energy distribution between liquid and solid food [25], we also stratified the energy contribution (%) obtained from both solid (solid calories) and liquid sources (liquid calories) in the estimation of caloric intake. Participants were asked to disclose any consumption of prescribed medication or dietary supplementation.

### Blood sample collection and processing

After 8–9 h of overnight fasting, blood samples were collected from all participants in the morning at all three collection timepoints. Venous blood (20 ml) were collected from an antecubital vein into Vacutainer tubes (BD Vacutainer, Franklin Lakes, NJ). Whole blood was used for white and red blood cell enumeration. Tubes were centrifuged for 10 min at 1200*g* to allow the collection of plasma. Blood tubes were allowed to clot for 1 h before centrifugation in the isolation of serum. Whole blood and plasma samples were analysed within 2 h while serum samples were stored at − 80 °C for shipment and further measurements.

### Biomarker study

Blood samples were tested for blood count (white blood cells, haemoglobin, platelets) at the Clinical and Research laboratory in Charsadda, KPK, Pakistan. Plasma samples were analysed for the levels of glucose, triglycerides, C-reactive protein, creatinine, ferritin and blood urea nitrogen (BUN) using a biochemical blood analyzer (Hitachi 7180, Hitachi, Japan). Cell counts for WBC (white blood cells), RBC (red blood cells), neutrophils, lymphocytes, monocytes, eosinophils, basophils, as well as haemoglobin concentration, HCT (haematocrit), MCV (mean corpuscular volume) and MPV (mean platelet volume) were obtained using a blood cell counter (Hemavet 0950, CDC Technology, USA). Serum from participants were thawed and adiponectin (Abcam, Cambridge, UK), ketone bodies (Cayman Chemical, MI, USA), orexin A (Wuhan USCN Business Co., Ltd., Hubei, China), PPARβ (MyBioSource, CA, USA) and sCD14 (Elabscience, Hubei, China) levels were measured by Enzyme-Linked Immunosorbent Assay (ELISA). Serum samples were added to 96-well plates that have been pre-coated with antibodies specific to the marker of interest. The bound markers were then incubated with enzyme-linked antibody conjugates. Wells were washed to remove any unbound antibodies. Substrate solution was added to the wells and the reaction was stopped when colour development was observed to be optimal, as per manufacturer’s recommendation. The optical densities were measured at wavelengths indicated in the manufacturers’ protocol on the EnVision^®^ 2104 multimode microplate reader (Perkin Elmer, MA, USA). All other biomarkers were measured using multiplex immunoassays, MILLIPLEX© Multiplex Assays (Merck Millipore, MA, USA) and ProcartaPlex multiplex panel (Thermo Fisher Scientific, MA, USA), based on Luminex^®^ xMAP^®^ technology. Serum samples were incubated overnight in 96-well plates with fluorescent-coded magnetic beads; each conjugated with capture antibodies against the marker of interest. The plates were washed and biotinylated detection antibodies were incubated with the complex for 1 h. Streptavidin-PE was then added and incubated for 30 min. The plates were washed and the beads were resuspended in sheath fluid for measurement on the Luminex™ FLEXMAP 3D^®^ (Luminex, TX, USA). Data was acquired using xPONENT^®^ 4.0 (Luminex, TX, USA) software and analyzed with the Bioplex Manager™ 6.0 software (Bio-Rad Laboratories, Hercules, CA, USA).

### Statistical analysis

The analysis of specific biomarkers was performed using Prism V8.0 (GraphPad). Violin plots reveal the medians, quartiles and the distribution of individual data and one-way ANOVA with repeated measures was used for group comparisons. Log- or square root transformation (depending on the obtained best fit to the Gaussian model) was employed when data were not normally distributed. Chi squared test was used in order to the categorical variables’ distribution. Regression analyses were performed using Stata 14 (Stata Corporation, College Station, Texas, 2015), α = 0.05. Results are presented as estimates with associated standard errors or 95% confidence intervals. All quantitative biological variables related to inflammation with left censored data were converted to categorical variables: (i) by using the limit of detection or LOD (0.64 pg/ml); or (ii) by using the mean or the median to obtain binary variables. The selected cut-off point depended on the variable distribution: (i) the detection limit was used if more than 50% of the observations fell below it; (ii) the median was used where the frequency of observations below the detection limit was > 25% and < 50%.

Finally, in order to appreciate shifting trends in biomarker concentrations during fasting, we used identical cut-off points to categorize data into three study phases. Specifically, the median computed during the pre-fasting phase were used to categorize data during the fasting and post-fasting phases. Only CRP, platelets, WBC, APRIL, MIG and calcitonin variables, with fully available observations, were considered as quantitative variables.

#### Categorical (binary) variables

A repeated measures logistic regression model (RML) was used to assess crude and adjusted associations between the plasma concentration of each biomarker (coded as 0/1 for < cut-off value and ≥ cut-off value) and the different stages of fasting, with the pre-fasting phase used as the reference point. The regression model was performed for each binary variable by using the ‘Xtgee’ STATA command and ‘logit’ link function, and based on a ‘binomial’ distribution.

#### Continuous variables

A generalized least squares (GLS) random-effects model was fitted to each variable using the ‘Xtreg’ STATA command.

Participants were analysed as a whole before further subdivision into younger (20–30 year old) and older adult (50–85 year old) age groups. For all regression models, crude and adjusted analyses were performed. Based on literature, age (only on the entire data) [26], body mass index [26] and energy intake [27] were identified as possible confounding factors. Since food quality has a profound influence on inflammatory levels, food intake was calibrated against their dietary inflammatory index (DII) score [28]. In order to remove confounder factors effect, we operated an adjustment for age, body mass index, energy intake and dietary inflammatory index (DII) score. The principal component analysis (PCA) was applied so as to identify components reflecting young or old adults associated profile or signature. In general, the components with eigenvalue an eigenvalue > 1 were retained. This was checked by a meticulous examination of the scree plot. With the intention of depicting the main contributors for each component, an absolute cut-off value for factor loadings (> |0.2|) was used.

## Results

### Participants, compliance and adverse events

In this prospective study, we followed the progress of adult men, who completed a daily average of 16 h fasting for 29 consecutive days. Women participants (n = 5) were not included in this analysis due to their paucity. Participants began fasting at sunrise and ended their fast at sunset, leaving a maximum of 8 h for food intake (Fig. 1a). The nutritional intake of participants was not restricted but the study area was selected based on trends and dietary behaviour from previous fasting seasons during which an average of 3.0 kg weight loss was observed. Questionnaires were collected in the presence of a household relative to corroborate the dietary report and participants were requested to resume their daily routine. All participants fasted simultaneously, starting and ending at a similar time of day; the elderly were not precluded so that we could study the effect of age. Blood was collected before the commencement of fasting, on Week 4 of fasting and 1 month after the end of fasting, for the measurement of cardiometabolic and inflammatory markers.

The basic baseline demographic characteristics of participants are shown in Table 1. All participants were recruited from semi-urban areas with few displaying chronic diseases, as this is often a medical condition preventing from fasting. For instance, only 7.6% have reported diabetes as a condition and while hypertension was not reported we assume a similar limited fraction of participants exhibit such condition. Most participants (80%) were free from adverse health events during the fasting period, while some suffered from diarrhoea (5.0%), fever (5.0%) or malaria (10%). Mean age was 47.1 ± 21.8 years—young (n = 37, 20 to 30 years old), middle-aged (n = 18, 52 to 64 years old) and elderly (n = 23, 65 to 83 years old). Although the educational level of participants was generally low, as more than 30% had not received formal education, a majority of participants (90%) had average monthly incomes (mean USD 340.8 ± 212.8). The average age when participants started annual long-term fasting was 11.5 ± 2.5 years. Five participants missed fasting for 2–3 days as they were travelling (n = 2) or ill (n = 3). Approximately one-third of participants (30.7%) fasted for several days (5.4 ± 1.6 days) in other periods of the year.

**Table 1:** Baseline demographic characteristics (n = 78)

Age	
Mean (± SD) in years	47.1 (± 21.8)
20 to 30 years (young)	37 (47.4%)
52 to 64 years (middle-aged)	18 (23.1%)
> 64 (elderly)	23 (29.5%)
Education	
No formal education	24 (30.7%)
Primary	43 (55.1)
Middle/high	8 (10.2%)
Higher secondary	5 (6.4%)
Nutritional status	
Thinness	19 (24.4%)
Normal	36 (46.2%)
Overweight	20 (25.6%)
Obesity	3 (3.8%)
Fasting habits	
Fast only in Ramadan	68 (69.2%)
Also fast other than Ramadan	24 (30.7%)
Monthly incomes	
Mean (± SD) in USD	340.8 (± 212.8)
> 500 USD/month	21 (26.9%)
200–500 USD/month	45 (57.7%)
< 200 USD/month	12 (15.4%)
Others	
Diabetes	6 (7.6%)
Marital status—married	67 (85.9%)
Smokers	32 (41.0%)
Snuff use	28 (35.9%)
Alcohol use	0 (0.0%)

### Dietary patterns and nutritional intake

Aside from small quantities of cooked food items that were purchased from the market, the bulk of consumed food was prepared at home (84%) thus mainly excluding the possibility of results affected by foods of non-home origin. The descriptions of consumed food and beverage, as well as average consumption during the fasting period are provided in Additional file 1: Tables S1, S2. Any significant deviation from the average dietary pattern was recorded, including information on the frequency and type of food. For example, participants had 3.4 ± 1.6 different types of dishes a day during non-fasting periods but this expanded to 12.5 ± 2.8 different dishes during the 29-day fasting period. The frequency of food intake, however, was higher (Fig. 1b) during non-fasting days as compared to fasting days (7.2 ± 1.2 vs. 4.2 ± 2.3 times per day, respectively). With less frequent but more diverse nutritional intake, fasting resulted in a significant reduction of caloric (p < 0.0001) and carbohydrate intake (p = 0.003), while the consumption of fat, proteins and fibre was maintained (Table 2).

**Table 2:** Assessment of basic nutritional and clinical status

Parameters	Pre-fasting	Fasting	Post-fasting	p-value
Nutritional status				
Calories (kcal/day)				
Mean (s.d.)	2009.5 (583.4)	1835.4 (584.6)	2020.6 (594.8)	< 0.0001
Median	2011.0	1805.8	1982.4	
Protein (g/day)				
Mean (s.d.)	41.8 (13.5)	40.8 (13.5)	41.9 (13.6)	< 0.0001
Median	39.9	39.0	39.9	
Fats (g/day)				
Mean (s.d.)	85.6 (45.2)	83.9 (45.2)	85.8 (45.8)	< 0.0001
Median	76.4	74.6	77.3	
Carbohydrates (g/day)				
Mean (s.d.)	263.3 (34.5)	224.6 (54.2)	219.4 (41.7)	0.003
Median	76.4	74.6	77.3	
Dietary fibers (g/day)				
Mean (s.d.)	5.4 (2.2)	5.2 (2.2)	5.6 (2.3)	< 0.0001
Median	5.0	5.0	5.4	
Sodium (g/day)				
Mean (s.d.)	2.9 (0.3)	3.1 (0.4)	3.1 (0.5)	< 0.0001
Median	2.8	3.0	3.2	
Potassium (mg/day)				
Mean (s.d.)	1737.2 (581.8)	1719.8 (576.0)	1754.5 (587.6)	< 0.0001
Median	1693.3	1676.4	1710.2	
Iron (g/d)				
Mean (s.d.)	13.8 (5.5)	12.9 (5.5)	14.9 (5.5)	< 0.0001
Median	13.2	12.3	14.3	
Clinical status				
Weight (kg)				
Mean (s.d.)	67.5 (15.0)	63.7 (14.8)	66.4 (14.8)	< 0.0001
Median	68.8	64.6	67.6	
BMI (kg/m^2^)				
Mean (s.d.)	22.8 (5.1)	21.6 (5.0)	22.5 (5.0)	< 0.0001
Median	23.5	22.3	23.2	
Waist circumf. (cm)				
Mean (s.d.)	85.3 (12.4)	84.4 (12.5)	84.4 (12.5)	< 0.0001
Median	88.1	86.7	0.9 (0.1)	
Albumin (g/l)				
Mean (s.d.)	3.9 (1.0)	3.7 (1.2)	3.7 (1.0)	0.0013
Median	3.8	3.4	3.6	
Ferritin (µg/l)				
Mean (s.d.)	75.5 (48.4)	77.2 (49.2)	74.6 (48.4)	0.0013
Median	73.4	73.6	71.9	
Systolic BP (mmHg)				
Mean (s.d.)	140.6 (25.9)	124.2 (22.9)	130.0 (22.6)	< 0.0001
Median	138.6	102.9	124.8	
Diastolic BP (mmHg)				
Mean (s.d.)	107.6 (24.5)	102.9 (25.6)	106.2 (24.8)	< 0.0001
Median	104.5	97.3	102.0	

### Physiological response to fasting

While TRF and FMD are not usually accompanied by weight loss, we observed a significant reduction in the weight of participants at the end of fasting (mean difference = − 3.69 kg ± 0.15), but weight recovery ensued in the following weeks (mean difference = + 2.60 kg ± 0.03). This matched our previous observations and also confirms the power of the study to be sufficient for the observation of > 3.0 kg weight loss. Although the extent of weight loss was heterogenous among subjects (in terms of absolute values), post-fasting weight gain was more consistent throughout the cohort (Fig. 1c). During the fasting period, we analysed metabolic markers (Fig. 1d) associated with nutrient sensing. Leptin, which promotes satiety, was significantly reduced during fasting (p = 0.0177) and post-fasting leptin levels remained significantly lower than baseline (p = 0.0102). Peroxisome proliferator-activated receptors (PPAR)-β, which is involved in lipid and glucose homeostasis and is the most abundant member of the PPAR family, was also reduced during (p = 0.0002) and post-fasting (p = 0.0052). Similarly, fasting glucose levels were significantly reduced during the fasting period (p < 0.0001) and the difference was sustained during the post-fasting period (p < 0.0001). A series of nutritional, clinical, and inflammatory markers are presented as a heat map in Additional file 2: Figure S1 to provide an overview of the shifts induced by fasting and any evidence of post-fasting recovery.

### Health benefits from fasting: lower blood pressure, heart rate and biomarkers of cardiovascular risk and metabolic status

**Fig. 2:**
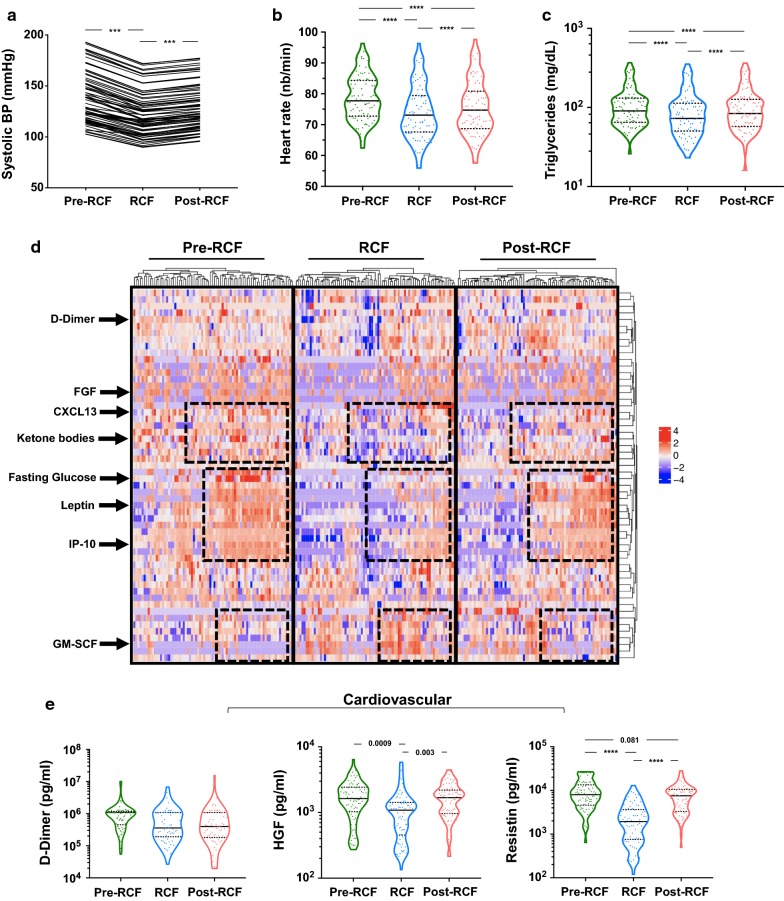

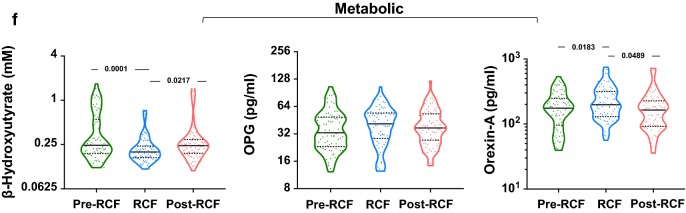
Metabolic adaptation to RCF. **a**–**c** Clinical laboratory measures suggesting beneficial effect of fasting. **d** Heatmap of biological markers of physiological functions measured during the course of the study. All biomarkers measured in plasma/serum samples are shown. Regions highlighted show the overall shift during fasting and return to baseline after fasting. **e** Specific biomarkers of cardiovascular and **f** metabolic status

As shown in Fig. 2a, systolic blood pressure was significantly reduced by fasting (140.6 mmHg ± 25.9 vs. 124.2 mmHg ± 22.9). This effect persisted in the post-fasting period as average systolic pressure remained at 130.0 mmHg ± 22.6 1 month after fasting (Table 2, p < 0.0001). Similar observations were made for diastolic blood pressure (Table 2) and heart rate (Fig. 2b, p < 0.0001). In the studied population, 33/78 (42.3%) individuals had high systolic BP but this was reduced to 19/78 during fasting. Triglyceride levels, which are positively associated with cardiovascular risks, were reduced during and after fasting (Fig. 2c).

We observed significant shifts in the levels of circulating biomarkers during and after fasting, as highlighted in dotted boxes in the heat map shown in Fig. 2d, which represents all the circulating biomarkers measured in the serum by ELISA/Multiplex. Specific biomarkers of interest related to cardiovascular and metabolic health are described in further detail in Fig. 2e, f, respectively. d-Dimer, the by-product of fibrin, reflects thrombus formation and lysis while hepatocyte growth factor (HGF), initially thought to be liver-specific, promotes angiogenesis and may play an important role in cardio-protection and post-injury cardiomyocyte regeneration [29]. Resistin acts in the pathogenesis of atherosclerosis by promoting endothelial dysfunction, vascular smooth muscle cell proliferation, arterial inflammation, and the formation of foam cells. Resistin is also an adipokine that is linked to insulin resistance. All three molecules are independent risk factors for cardiovascular diseases [30]. While not significant (p = 0.075), likely due to heterogeneous readings, a 30% reduction in average d-dimer levels was observed during the fasting period. Nevertheless, fasting induced a significant reduction in HGF (p = 0.0009) and resistin (p < 0.0001) levels, although both were restored to baseline levels after fasting.

As liquid intake was prohibited during the 16 h fasting period, we also analysed kidney function by measuring blood urea nitrogen (BUN) and creatinine levels. As expected, both metabolites were significantly lowered by fasting (Additional file 2: Figure S2A, p < 0.0001) and the BUN-to-creatinine ratio before, during and after fasting was 14.55 ± 2.33, 16.25 ± 4.11 and 14.07 ± 1.63 respectively (p < 0.0001). As for the assessment of liver function, we observed significant changes in l-alanine aminotransferase (ALAT) and l-aspartate aminotransferase (ASAT) levels during fasting (p < 0.0001, data not shown), in addition to previously described changes in HGF levels.

As our initial analysis revealed prolonged glycemia during and after fasting, we were interested in the levels of β-hydroxybutyrate, which could be triggered as a compensatory mechanism to promote ketosis. On the contrary, we observed that fasting contributed to reduced β-hydroxybutyrate levels (Fig. 2f). In investigating the potential benefits of fasting, we tested metabolites associated with bone health. Levels of osteoprotegerin (OPG), which inhibits osteoclastogenesis and bone resorption, were increased but this difference did not reach statistical significance. Nevertheless, we observed a robust elevation in the levels of orexin-A (p = 0.0183), a hypothalamus-derived hormone which promotes muscle toning and wakefulness. Finally, we were interested in whether there were any fasting-induced changes in gut-permeability and measured levels of sCD14—a surrogate marker of microbial translocation. sCD14 levels were significantly increased during fasting (Additional file 2: Figure S2, p = 0.0422), which suggests reduced barrier function and more bacterial translocation.

### Recurrent fasting and inflammation

**Fig. 3:**
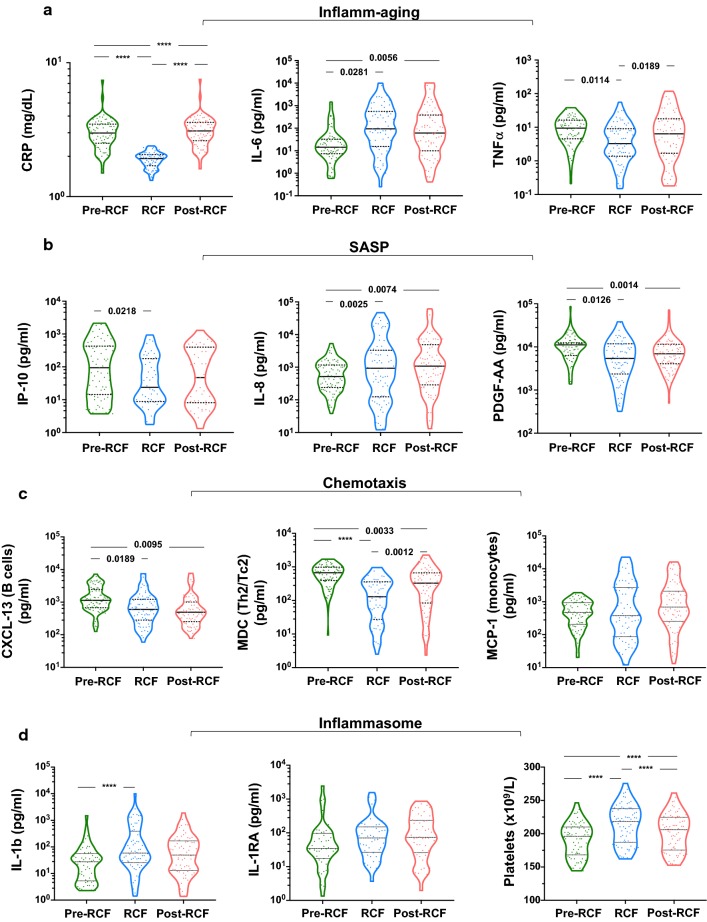
Systemic inflammation in response to RCF. **a** Biomarkers of inflammaging, **b** senescence-associated secretory profile (SASP), **c** chemotaxis of immune cells and **d** inflammasome measured in serum of participants before, during and after completion of RCF

Figure 3a shows a comparison of the levels of inflammatory cytokines at all three time points. All participants had CRP levels below 3 mg/ml during the fasting period (1.89 ± 0.25 mg/ml), but post-fasting levels of CRP were slightly elevated above baseline (3.14 ± 0.84 mg/ml); the number of individuals with elevated post-fasting CRP levels was similar to the number observed before fasting. While a similar profile was observed for TNFα, IL-6 levels were elevated (p = 0.0281) during fasting and remained elevated thereafter (p = 0.0056). Using the Chi squared test, multinomial regression and linear regression, we further probed the evolution of CRP, IL-6 and TNFα during the different stages of fasting (Table 3). Plasma TNFα concentration was significantly reduced during (− 26.3%, p = 0.001) and after fasting (− 9.6%, p = 0.003). Similarly, CRP levels were significantly altered across the three study points (p < 0.0001). Data from regression (GLS and RMRL) models indicate that fasting promotes a reduction of TNF-α levels (coef. = − 1.16; 95% CI [− 1.91 to − 0.42]) but increases IL-6 levels (coef. = + 0.77; 95% CI [0.06 to 1.49]). After eliminating confounding factors, only older adults (aged ≥ 52 year) were resistant to elevations in TNFα levels (coef. = − 1.56; 95% CI [− 2.61 to − 0.52]) during fasting. Younger adults were more resilient to increased plasma TNFα levels in the post-fasting phase (adjusted coef. = − 1.35; 95% CI [− 2.52 to − 0.18]). While fasting was associated with a significant decrease in CRP levels (− 1.14 mg/L) across all participants, age was associated with greater CRP decrease (− 1.61 vs. − 0.65 mg/L; p < 0.0001).

**Table 3:** Assessment of fasting on plasma pro-inflammatory biomarkers and their evolution during post-fasting phase (n = 78)

	Crude data analysis	Repeated measures logistic regression and generalized least squares random-effects model											
		All				Young (20–30 years)				Adult-to-elderly (≥ 52 years)			
	Diff.^1^ (% ≥ cut-off value) or mean (s.d.)	Crude coeff.^2^	95% CI^3^	Adjusted Coeff.^4^	95% CI^3^	Crude coeff.^2^	95% CI^3^	Adjusted Coeff.^4^	95% CI^3^	Crude coeff.^2^	95% CI^3^	Adjusted Coeff.^4^	95% CI^3^
	IL-6												
	*P*^5^ = 0.77	*P*^6^ = 0.091		*P*^7^ = 0.074		*P*^6^ = 0.54		*P*^7^ = 0.45		*P*^6^ = 0.10		*P*^7^ = 0.075	
Pre-fasting	–	1		1		1		1		1		1	
Fasting	+ 15.4	0.64	− 0.03 to 1.30	+ 0.77	0.06 to 1.49	+ 0.54	− 0.42 to 1.51	+ 0.65	− 0.41 to 1.71	+ 0.74	− 0.19 to + 1.66	+ 0.89	− 0.17 to 1.95
Post-Fasting	+ 15.4	0.63	− 0.03 to 1.30	+0.58	− 0.11 to 1.29	+ 0.32	− 0.64 to 1.29	+ 0.14	− 0.91 to 1.17	+ 0.98	*0.02 to 1.94*	+ 1.11	0.05 to 2.18

	TNF-α												
	*P*^*5*^= 0.003	*P*^6^ = 0.0029		*P*^7^ = 0089		*P*^6^ = 0.038		*P*^7^ = 0.044		*P*^6^ = 0.0039		*P*^7^ = 0.0021	
Pre-fasting	–	1		1		1		1		1		1	
Fasting	− 26.3	− 1.17	− 1.85 to − 0.50	− 1.16	− 1.91 to − 0.42	− 1.06	− 2.09 to − 0.02	− 1.03	− 2.15 to 0.09	− 1.27	− 2.19 to − 0.35	− 1.56	− 2.61 to − 0.52
Post-fasting	− 9.6	− 0.39	− 1.01 to 0.23	− 0.35	− 1.03 to 0.33	− 1.22	− 2.30 to − 0.16	− 1.35	− 2.52 to 0.18	+ 0.20	− 0.65 to 1.05	+ 0.50	− 0.44 to1.44

	C-reactive protein												
	*P*^5^ < 0.0001	*P*^6^ < 0.0001		*P*^7^ < 0.0001		*P*^6^ < 0.0001		*P*^7^ < 0.0001		*P*^6^ < 0.0001		*P*^7^ < 0.0001	
Pre-fasting	3.03 (0.84)	1		1		1		1		1		1	
Fasting	1.89 (0.25)	− 1.14	− 1.30 to − 0.97	− 1.14	− 1.31 to − 0.96	− 0.62	− 0.76 to −0.47	− 0.65	− 0.81 to − 0.50	− 1.61	− 1.83 to − 1.39	− 1.62	− 1.88 to − 1.36
Post-fasting	3.14 (0.84)	+ 0.11	− 0.05 – 0.27	+ 0.11	− 0.07 to 0.28	+ 0.11	− 0.03 to 0.25	+ 0.15	− 0.01 to 0.31	+ 0.11	− 0.11 to 0.33	+ 0.14	− 0.11 to 0.38

^1^Difference (between pre-fasting vs. fasting phases and pre-fasting vs. post-fasting phases) in percentage terms of biomarker concentration ≥ cut-off value or mean ± standard deviation

^2^Crude generalized least squares random-effects model or repeated measures logistic regression model to examine the association between biomarkers level with fasting or not state

^3^C.I. 0.95 confidence interval for Diff

^4^Adjusted generalized least squares random-effects model or Repeated measures logistic regression model to examine the association between biomarkers level with fasting or not state

^5^p-values: null hypothesis of identical percentage differences of each biomarker concentration level for the pre-, per- and post-fasting or equality of means

^6^Crude p-value for association of biomarker plasma concentration ≥ cut-off value or for association difference of means for interval variables with co-factor

^7^Adjusted p-value for association of biomarker plasma concentration ≥ cut-off value or for association difference of means for interval variables with co-factor

In consideration of our observations on inflammatory markers, we further tested whether molecules composite to the senescence-associated secretory phenotype (SASP) were also similarly regulated by fasting (Fig. 3b). Here, we show that IP-10 (interferon gamma-induced protein 10) and platelet-derived growth factor AA (PDGF-AA) were significantly downregulated following recurrent fasting (p = 0.0218 and p = 0.0126, respectively). However, IL-8 levels were increased during fasting (p = 0.0025) and remained high after the fasting period, similar to IL-6. Altogether, the data suggest an complex regulation of inflammatory processes during RCF. Investigating deeper, we tested for any modulation in the circulating levels of chemokines related to three immune cell populations: T helper cells, B cells and monocytes (Fig. 3c). The data suggest an overall diminution in the expression of chemokines during fasting. Based on this data we finally intended to show a potential involvement of a key regulator of inflammation, the inflammasome (Fig. 3d). IL-1β, a core molecule of the inflammasome, was significantly increased by fasting (263.3 ± 1383 vs. 674.9 ± 2079, p < 0.0001). Platelets, a significant source of inflammation showed a concomitant increase in their numbers (p < 0.0001). IL-1β signalling is known to be counteracted by circulating IL1-RA, which binds competitively to IL-1R. Nevertheless, we did not observe a significant increase in IL1-RA levels in our paired analysis.

### Associations and regression models

For a more holistic approach, we performed two types of statistical analysis on participant data during the three fasting phases. Table 4 shows the different associations between the levels of biomarkers with fasting status.

**Table 4:** Assessment of fasting on plasma biomarkers and their evolution during post-fasting phase (n = 78)

	Crude data analysis	Repeated measures logistic regression and generalized least squares random-effects model											
		Total				Young (20–30 years)				Adult-to-elderly (≥ 52 years)			
	Diff.^1^ (% ≥ cut-off value) or mean (s.d.)	Crude coeff.^2^	95% CI^3^	Adjusted coeff.^4^	95% CI^3^	Crude coeff.^2^	95% CI^3^	Adjusted coeff.^4^	95% CI^3^	Crude Coeff.^2^	95% CI^3^	Adjusted coeff.^4^	95% CI^3^
Haematological markers													
	Platelets												
	*P*^*5*^< 0.0001	*P*^6^ < 0.0001		*P*^7^ < 0.0001		*P*^6^ < 0.0001		*P*^7^ < 0.0001		*P*^6^ < 0.0001		*P*^7^ < 0.0001	
Pre-fasting	190.7 (24.8)	1		1		1		1		1			
Fasting	213.5 (28.8)	+ 22.8	21.9 to 23.7	+ 27.66	26.17 to 29.15	+ 22.95	21.68 to 24.21	+ 28.57	26.43 to 30.72	+ 22.73	21.5 to 24.0	+ 27.29	25.23 to 29.34
Post-Fasting	201.6 (27.7)	+ 10.9	10.0 to 11.8	+ 11.71	10.55 to 12.87	+ 10.95	9.96 to 12.21	+ 12.88	11.06 to 14.70	+ 10.87	9.63 to 12.11	+ 10.74	9.21 to 12.27

	WBC												
	*P*^5^ < 0.0001	*P*^6^ < 0.0001		*P*^7^ < 0.0001		*P*^6^ = 0.0002		*P*^7^ = 0.0001		*P*^6^ < 0.0001		*P*^7^ < 0.0001	
Pre-fasting	5.68 (0.70)	1		1		1		1		1		1	
Fasting	6.23 (0.75)	+ 0.55	0.34 to 0.75	+ 0.62	0.39 to 0.85	+0.45	0.21 to 0.70	+ 0.56	0.30 to 0.83	+ 0.63	0.31 to 0.96	+ 0.70	0.32 to 1.05
Post-fasting	5.55 (1.32)	+ 0.13	− 0.33 to 0.08	− 0.06	− 0.29 to 0.16	+ 0.02	− 0.22 to 0.26	+ 0.05	− 0.21 to 0.32	− 0.26	− 0.58 to 0.06	− 0.22	− 0.57 to 0.13

Inflammation biomarkers													
Interleukines: *Common b chain* (*CD131*)													
	IL-7												
	*P*^5^ = 0.30	*P*^6^ = 0.018		*P*^7^ = 0.045		*P*^6^ = 0.32		*P*^7^ = 0.29		*P*^6^ = 0.066		*P*^7^ = 0.0.094	
Pre-fasting	–	1		1		1		1		1		1	
Fasting	− 12.8	− 1.24	− 2.21 to − 0.28	− 0.95	− 2.05 to 0.14	− 0.65	− 1.68 to 0.37	− 0.56	− 1.75 to 0.62	− 2.27	− 4.46 to − 0.08	− 2.07	− 4.38 to 0.23
Post-fasting	− 10.3	− 0.88	− 1.74 to − 0.02	− 1.08	− 2.09 to − 0.06	− 0.65	− 1.68 to 0.37	− 0.91	− 2.15 to 0.32	− 1.12	− 2.58 to 0.33	− 1.16	− 2.75 to 0.43

	IL-9												
	*P*^5^ = 0.16	*P*^6^ = 0.24		*P*^7^ = 0.33		*P*^6^ = 0.48		*P*^7^ = 0.61		–		*P*^7^ = 0.72	
Pre-fasting	–	1		1		1	–	1				1	
Fasting	− 2.6	− 0.43	− 1.76 to 0.90	− 0.56	− 2.00 to 0.87	− 0.57	− 2.16 to 1.02	− 0.45	− 2.15 to 1.26	–	–	− 0.84	− 3.95 to 2.27
Post-fasting	− 6.4	− 1.85	− 4.03 to 0.31	− 1.57	− 3.76 to 0.63	0.00	–	0.00	–	–	–	+ 0.51	− 2.59 to 3.62

	GM-CSF												
	*P*^5^ < 0.0001	*P*^6^ = 0.0001		*P*^7^ = 0.0001		*P*^6^ = 0.025		*P*^7^ = 0.016		*P*^6^ = 0.0022		*P*^7^ = 0.0024	
Pre-fasting	–	1		1		1		1		1		1	
Fasting	+ 35.9	+ 1.57	0.85 to 2.28	+ 1.65	0.90 to 2.41	+ 1.37	0.36 to 2.39	+ 1.52	0.43 to 2.61	+ 1.76	0.75 to 2.77	+ 1.86	0.75 to 2.97
Post-fasting	+ 20.5	+ 0.94	0.23 to 1.65	+ 0.87	0.14 to 1.61	+ 0.49	− 0.52 to 1.51	+ 0.28	− 0.79 to 1.37	+ 1.36	0.36 to 2.37	+ 1.41	0.34 to 2.49

Interleukines-*IL*-*10*-*like*													
	IL-10												
	*P*^5^ = 0.082	*P*^6^ = 0.039		*P*^7^ = 0.12		*P*^6^ = 0.21		*P*^7^ = 0.33		*P*^6^ = 0.17		*P*^7^ = 0.33	
Pre-fasting	–	1		1		1		1		1		1	
Fasting	− 15.5	− 0.85	− 1.52 to − 0.19	− 0.70	− 1.44 to 0.04	− 1.14	− 2.43 to 0.15	− 1.00	− 2.38 to 0.39	− 0.78	− 1.61 to 0.04	− 0.65	− 1.60 to 0.29
Post-fasting	− 6.4	− 0.31	− 0.91 to 0.28	− 0.44	− 1.11 to 0.23	− 0.17	− 1.19 to 0.86	− 0.49	− 1.69 to 0.71	− 0.42	− 1.21 to 0.37	− 0.40	− 1.28 to 0.47

Interleukines: *IL*-*1*-*like*													
	IL-1α												
	*P*^5^ < 0.0001	*P*^6^ < 0.0001		*P*^7^ < 0.0001		*P*^6^ = 0.0003		*P*^7^ = 0.0005		*P*^6^ < 0.0001		*P*^7^ < 0.0001	
Pre-fasting	–	1		1		1		1		1		1	
Fasting	+ 53.8	+ 2.61	1.79 to 3.42	+ 2.90	1.98 to 3.82	+ 1.90	0.90 to 2.89	+ 2.23	1.07 to 3.39	+ 3.73	2.08 to 5.39	+ 4.01	2.22 to 5.80
Post-fasting	+ 48.7	+ 2.38	1.57 to 3.19	+ 2.32	1.45 to 3.19	+ 1.67	0.68 to 2.65	+ 1.61	0.50 to 2.72	+ 3.52	1.87 to 5.16	+ 3.47	1.76 to 5.18

	IL-1 β												
	*P*^5^ = 0.037	*P*^6^ = 0.036		*P*^7^ = 0.041		*P*^6^ = 0.52		*P*^7^ = 0.39		*P*^6^ = 0.039		*P*^7^ = 0.061	
Pre-fasting	–	1		1		1		1		1		1	
Fasting	+ 14.1	+ 0.76	0.03 to 1.50	+ 0.65	− 0.13 to 1.45	+0.27	− 0.69 to 1.23	+ 0.32	− 0.78 to 1.42	+ 1.32	0.14 to 2.48	+ 1.15	− 0.12 to 2.44
Post-fasting	+ 15.4	+ 0.85	0.11 to 1.60	+ 0.95	0.15 to 1.76	+ 0.60	− 0.42 to 1.60	+ 0.83	− 0.35 to 2.01	+ 1.10	− 0.01 to 2.22	+ 1.17	− 0.04 to 2.38

Interleukines: *IL*-*6*-*like*													
	IL-12P40												
	*P*^5^ = 0.44	*P*^6^ = 0.38		*P*^7^ = 0.086		*P*^6^ = 0.50		*P*^7^ = 0.18		*P*^6^ = 0.32		*P*^7^ = 0.22	
Pre-fasting	–	1		1		1		1		1		1	
Fasting	+ 6.4	+ 0.67	− 0.28 to 1.64	+ 1.17	0.05 to 2.31	+ 0.40	− 0.66 to 1.46	+ 0.70	− 0.58 to 2.00	+ 1.46	− 0.84 to 3.77	+ 2.27	− 0.30 to 4.85
Post-Fasting	+ 3.8	+ 0.45	− 0.55 to 1.44	+ 0.09	− 1.04 to 1.22	− 0.25	− 1.44 to 0.93	+ 0.74	− 2.21 to 0.72	+ 1.71	− 0.54 to 3.97	+ 1.55	− 0.85 to 3.95

	IL-12P70												
	*P*^5^ = 0.069	*P*^6^ = 0.11		*P*^7^ = 0.16		*P*^6^ = 0.13		*P*^7^ = 0.28		*P*^6^ = 0.79		*P*^7^ = 0.95	
Pre-fasting	–	1		1		1		1		1		1	
Fasting	− 5.1	− 0.90	− 2.26 to 0.46	− 0.58	− 2.17 to 1.00	− 1.72	− 3.98 to 0.52	− 1.35	− 3.80 to 1.10	0.00	− 1.86 to 1.86	− 0.26	− 2.90 to 2.38
Post-fasting	− 7.7	− 2.03	− 4.11 to 0.065	− 2.07	− 4.29 to 0.14	0.00	–	0.00	–	− 0.72	− 2.99 to 1.56	− 0.37	− 3.19 to 2.44

Interleukines: *interferons*													
	IFN-Gamma												
	*P*^5^ = 0.068	*P*^6^ = 0.034		*P*^7^ = 0.051		*P*^6^ = 0.26		*P*^7^ = 0.33		*P*^6^ = 0.067		*P*^7^ = 0.14	
Pre-fasting	–	1		1		1		1		1		1	
Fasting	− 16.7	− 0.74	− 1.33 to − 0.14	− 0.74	− 1.39 to − 0.084	− 0.47	− 1.33 to 0.37	− 0.52	− 1.44 to 0.39	− 0.98	− 1.81 to − 0.15	− 0.94	− 1.92 to 0.03
Post-fasting	− 14.1	− 0.63	− 1.23 to − 0.038	− 0.60	− 1.23 to 0.04	− 0.69	− 1.54 to 0.15	− 0.62	− 1.54 to 0.28	− 0.58	− 1.42 to 0.26	− 0.58	− 1.50 to 0.35

Other interleukines													
	Il-17												
	*P*^5^ < 0.0001	*P*^6^ < 0.0001		*P*^7^ < 0.0001		*P*^6^ = 0.0009		*P*^7^ = 0.0014		*P*^6^ = 0.010		*P*^7^ = 0.014	
Pre-fasting	–	1		1		1		1		1		1	
Fasting	− 33.3	− 1.41	− 2.03 to − 0.79	− 1.26	− 1.92 to − 0.60	− 1.63	− 2.57 to − 0.69	− 1.51	− 2.48 to − 0.53	− 1.22	− 2.06 to − 0.39	− 1.10	− 2.03 to − 0.17
Post-fasting	*− *26.9	− 1.11	− 1.71 to − 0.50	− 1.25	− 1.89 to 0.59	− 1.35	− 2.26 to − 0.44	− 1.47	− 2.45 to − 0.49	− 0.89	− 1.70 to − 0.08	− 1.04	− 1.93 to − 0.16

TGF-β													
	FlT-3L												
	*P*^5^ < 0.0001	*P*^6^ = 0.0003		*P*^7^ = 0.0004		*P*^6^ = 0.020		*P*^7^ = 0.025		*P*^6^ = 0.020		*P*^7^ = 0.024	
Pre-fasting	–	1		1		1		1		1		1	
Fasting	+ 28.2	+ 2.46	1.22 to 3.71	+ 2.56	1.27 to 3.84	+ 2.00	0.47 to 3.53	+ 2.23	0.57 to 3.89	+ 3.03	0.89 to 5.16	+ 3.00	0.82 to 5.18
Post-fasting	+ 26.9	+ 2.41	1.16 to 3.65	+ 2.31	1.05 to 3.58	+ 2.12	0.60 to 3.65	+ 2.02	0.38 to 3.66	+ 2.81	0.66 to 4.95	+ 2.76	0.60 to 4.92

	TGF-α												
	*P*^5^ < 0.0001	*P*^6^ = 0.		*P*^7^ < 0.0001		*P*^6^ < 0.0001		*P*^7^ = 0.0001		*P*^6^ < 0.0001		*P*^7^ < 0.0001	
Pre-fasting	–	1		1		1		1		1		1	
Fasting	− 53.8	− 2.51	− 3.25 to − 1.77	− 2.56	− 3.35 to − 1.76	− 2.18	− 3.24 to − 1.13	− 2.26	− 3.38 to − 1.15	− 2.85	− 3.91 to − 1.79	− 3.07	− 4.28 to − 1.86
Post-fasting	− 26.9	− 1.12	− 1.12 to − 0.49	− 1.16	− 1.82 to − 0.49	− 1.72	− 2.70 to − 0.74	− 1.64	− 2.67 to − 0.62	− 0.64	− 1.47 to 0.20	− 0.68	− 1.60 to 0.23

TNF-α													
	sCD40l												
	*P*^5^ = 0.014	*P*^6^ = 0.0075		*P*^7^ = 0.0048		*P*^6^ = 0.021		*P*^7^ = 0.037		*P*^6^ = 0.23		*P*^7^ = 0.10	
Pre-fasting	–	1		1		1		1		1		1	
Fasting	− 1.28	− 0.05	− 0.65 to 0.53	+ 0.09	− 0.56 to 0.75	− 0.22	− 1.07 to 0.62	− 0.20	− 1.11 to 0.71	+ 0.11	− 0.72 to 0.94	+ 0.41	− 0.62 to 1.42
Post-fasting	− 19.2	− 1.00	− 1.67 to − 0.32	− 1.12	− 1.85 to − 0.40	− 1.37	− 2.36 to − 0.37	− 1.36	− 2.43 to − 0.31	− 0.64	− 1.58 to 0.27	− 0.90	− 1.94 to 0.13

	APRIL												
	*P*^5^ = 0.029	*P*^6^ = 0.26		*P*^7^ = 0.036		*P*^6^ = 0.019		*P*^7^ = 0.031		*P*^6^ = 0.71		*P*^7^ = 0.79	
Pre-fasting	4451.4 (6944.6)	1		1		1		1		1		1	
Fasting	3158.7 (5937.8)	− 1292.7	− 2914.3 to 328.9	− 984.8	− 2860.6 to 711.1	− 2550.7	− 5282.6 to 181.3	− 2032.1	− 4852.7 to 788.4	− 157.5	− 1980.5 to 1665.6	− 179.9	− 2150.7 to 1790.8
Post-fasting	2235.3 (2245.9)	− 2216.1	− 3837.7 to − 594.6	− 2204.2	− 3883.8 to − 524.6	− 3863.8	6595.7 to − 1131.9	− 3792.8	− 6635.4 to − 950.2	− 729.2	− 2552.3 to 1093.8	− 658.9	− 2850.3 to 1262.3

Chemokines: CC chemokines													
	MIP-1 alpha												
	*P*^5^ < 0.0001	*P*^6^ < 0.0001		*P*^7^ < 0.0001		*P*^6^ = 0.0003		*P*^7^ = 0. 0016		*P*^6^ < 0.0001		*P*^7^ = 0.0003	
Pre-fasting	–	1		1		1		1		1		1	
Fasting	− 46.2	− 2.04	− 2.77 to − 1.31	− 1.93	− 2.75 to − 1.13	− 2.12	− 3.17 to − 1.08	− 2.06	− 3.20 to − 0.92	− 2.02	− 3.05 to − 1.00	− 1.98	− 3.18 to − 0.78
Post-fasting	− 6.4	− 0.27	− 0.93 to 0.38	− 0.28	− 1.00 to 0.44	− 1.15	− 2.13 to − 0.18	− 1.19	− 2.28 to − 0.11	+ 0.55	− 0.37 to 1.48	+ 0.68	− 0.38 to 1.74

	MCP-2												
	*P*^5^ < 0.0001	*P*^6^ < 0.0001		*P*^7^ = 0.0001		*P*^6^ < 0.0001		*P*^7^ = 0.0001		*P*^6^ = 0. 0063		*P*^7^ = 0.044	
Pre-fasting	–	1		1		1		1		1		1	
Fasting	− 39.7	− 1.68	− 2.36 to − 1.00	− 1.58	− 2.32 to − 0.84	− 2.18	− 3.24 to − 1.12	− 2.26	− 3.45 to − 1.07	− 1.31	− 2.21 to − 0.41	− 1.25	− 2.32 to − 0.19
Post-fasting	*− *24.4	− 1.03	− 1.69 to − 0.37	− 1.09	− 1.81 to − 0.37	− 2.18	− 3.24 to − 1.12	− 2.34	− 3.54 to − 1.14	− 0.10	− 0.98 to 0.77	− 0.02	− 1.04 to 1.00

	MDC												
	*P*^5^ < 0.0001	*P*^6^ < 0.0001		*P*^7^ < 0.0001		*P*^6^ = 0.0010		*P*^7^ = 0.0007		*P*^6^ = 0.0007		*P*^7^ = 0.0008	
Pre-fasting	–	1		1		1		1		1		1	
Fasting	− 42.3	− 2.48	− 3.42 to − 1.54	− 2.60	− 3.60 to − 1.60	− 1.91	− 3.05 to − 0.76	− 2.01	− 3.2 to − 0.81	− 3.64	− 5.71 to − 1.56	− 4.00	− 6.18 to − 1.79
Post-fasting	− 28.2	− 1.28	− 1.97 to − 0.58	− 1.23	− 1.97 to − 0.49	− 1.51	− 2.55 to − 0.46	− 1.59	− 2.71 to − 0.48	− 1.08	− 2.02 to − 0.14	− 0.91	− 1.95 to 0.12

	Rantes												
	*P*^5^ = 0.066	*P*^6^ = 0.073		*P*^7^ = 0.20		*P*^6^ = 0.23		*P*^7^ = 0.27		*P*^6^ = 0.074		*P*^7^ = 0.18	
Pre-fasting	–	1		1		1		1		1		1	
Fasting	− 19.2	− 0.81	− 1.50 to − 0.11	− 0.64	− 1.39 to 0.09	− 0.68	− 1.78 to 0.41	− 0.48	− 1.65 to 0.68	− 0.87	− 1.76 to 0.02	− 0.81	− 1.81 to 0.18
Post-fasting	− 10.3	− 0.42	− 1.09 to 0.25	− 0.42	− 1.13 to 0.28	− 0.94	− 2.06 to 0.18	− 0.97	− 2.16 to 0.21	+ 0.06	− 0.79 to 0.91	+ 0.15	− 0.77 to 1.07

	GRO												
	*P*^5^ < 0.0001	*P*^6^ < 0.0001		*P*^7^ < 0.0001		*P*^6^ = 0.0002		*P*^7^ = 0.0004		*P*^6^ = 0.0095		*P*^7^ = 0.0058	
Pre-fasting	–	1		1		1		1		1		1	
Fasting	− 36.5	− 1.88	− 2.65 to − 1.12	− 2.02	− 2.86 to − 1.18	− 1.95	− 2.94 to − 0.95	− 1.94	− 3.01 to − 0.88	− 2.02	− 3.32 to − 0.72	− 2.32	− 3.75 to − 0.91
Post-fasting	− 22.4	− 0.97	− 1.60 to − 0.33	− 0.93	− 1.63 to − 0.22	− 1.48	− 2.41 to − 0.56	− 1.46	− 2.46 to − 0.46	− 0.48	− 1.40 to 0.42	− 0.33	− 1.33 to 0.66

	Eotaxin												
	*P*^5^ = 0.002	*P*^6^ = 0.0017		*P*^7^ = 0.0040		*P*^6^ = 0.018		*P*^7^ = 0.018		*P*^6^ = 0.068		*P*^7^ = 0.13	
Pre-fasting	–	1		1		1		1		1		1	
Fasting	− 25.6	− 1.15	− 1.82 to − 1.48	− 1.11	− 1.84 to − 0.37	− 1.15	− 2.05 to − 0.26	− 1.19	− 2.17 to − 0.21	− 1.17	− 2.17 to − 0.16	− 1.12	− 2.25 to 0.01
Post-fasting	− 19.2	− 0.82	− 1.46 to − 0.18	− 0.85	− 1.55 to − 0.15	− 1.02	− 1.90 to − 0.14	− 1.18	− 2.17 to − 0.18	− 0.63	− 1.56 to 0.29	− 0.58	− 1.61 to 0.46

Chemokines: CXC chemokines													
	ENA 78												
	*P*^5^ < 0.0001	*P*^6^ = 0.0004		*P*^7^ = 0.0016		*P*^6^ = 0.0047		*P*^7^ = 0.0099		*P*^6^ = 0.0080		*P*^7^ = 0.029	
Pre-fasting	–	1		1		1		1		1		1	
Fasting	− 29.5	− 1.35	− 2.03 to − 0.67	− 1.35	− 2.10 to − 0.61	− 1.55	− 2.52 to − 0.58	− 1.61	− 2.70 to − 0.53	− 1.17	− 2.12 to − 0.22	− 1.19	− 2.28 to − 0.11
Post-fasting	− 7.7	− 0.31	− 0.92 to 0.29	− 0.28	− 0.95 to 0.38	− 1.00	− 1.91 to − 0.10	− 0.94	− 1.95 to 0.069	+ 0.29	− 0.54 to 1.12	+ 0.39	− 0.55 to 1.33

	CXCL13												
	*P*^5^ < 0.0001	*P*^6^ = 0.0001		*P*^7^ = 0.0003		*P*^6^ = 0.0005		*P*^7^ = 0.0008		*P*^6^ = 0.018		*P*^7^ = 0.014	
Pre-fasting	–	1		1		1		1		1		1	
Fasting	− 20.5	− 0.87	− 1.46 to − 0.27	− 1.00	− 1.68 to − 0.31	− 0.55	− 1.33 to 0.21	− 0.60	− 1.45 to 0.25	− 1.18	− 2.06 to − 0.29	− 1.62	− 2.72 to − 0.52
Post-fasting	− 29.5	− 1.35	− 2.00 to − 0.71	− 1.26	− 1.96 to − 0.57	− 2.05	− 3.08 to − 1.02	− 2.11	− 3.21 to − 1.00	− 0.93	− 1.78 to − 0.07	− 0.64	− 1.63 to 0.34

	MIG												
	*P*^5^ < 0.0001	*P*^6^ < 0.0001		*P*^7^ < 0.0001		*P*^6^ = 0.0010		*P*^7^ = 0.0017		*P*^6^ = 0.0004		*P*^7^ = 0.0011	
Pre-fasting	2452.4 (3085.0)	1		1		1		1		1		1	
Fasting	719.4 (1277.2)	− 1733.0	− 2406.0 to − 1059.8	− 1762.9	− 2473.7 to − 1052.1	− 1957.1	− 3079 to − 834.5	− 1977.4	− 3143.1 to − 811.8	− 1530.7	− 2305.2 to − 756.3	− 1597.1	− 2540.2 to − 744.0
Post-fasting	1409.9 (2213.9)	− 1042.0	− 1715 to − 369.2	− 945.5	− 1648.4 to − 242.6	− 1696.4	− 2819.1 to − 573.8	− 1542.9	− 2718.0 to − 367.7	− 452.3	− 452.3 to − 1226.7	− 338.3	− 1162.8 to 486.2

	IP-10												
	*P*^5^ = 0.001	*P*^6^ = 0.0003		*P*^7^ = 0. 0006		*P*^6^ = 0.0009		*P*^7^ = 0.0022		*P*^6^ = 0.032		*P*^7^ = 0.039	
Pre-fasting	–	1		1		1		1		1		1	
Fasting	− 28.2	− 1.28	− 1.92 to − 0.63	− 1.30	− 2.01 to − 0.60	− 1.26	− 2.08 to − 0.44	− 1.27	− 2.15 to − 0.38	− 1.33	− 2.34 to − 0.32	− 1.47	− 2.62 to − 0.32
Post-fasting	− 19.2	− 0.81	− 0.81 to − 1.41	− 0.76	− 1.41 to − 0.11	− 1.40	− 2.24 to − 0.57	− 1.28	− 2.17 to − 0.39	− 0.30	− 1.18 to 0.57	− 0.21	− 1.19 to 0.77

Chemokines: CX3C chemokines													
	Fractalkine												
	*P*^5^ = 0.002	*P*^6^ = 0.0031		*P*^7^ = 0.023		*P*^6^ = 0.070		*P*^7^ = 0.078		*P*^6^ = 0.033		*P*^7^ = 0.095	
Pre-fasting	–	1		1		1		1		1		1	
Fasting	− 26.9	− 1.12	− 1.79 to − 0.46	− 1.00	− 1.70 to − 0.28	− 1.02	− 1.89 to − 0.15	− 1.07	− 2.03 to − 0.12	− 1.22	− 2.22 to − 0.22	− 1.13	− 2.21 to − 0.05
Post-fasting	− 9.00	− 0.40	− 1.07 to 0.27	− 0.44	− 1.15 to 0.26	− 0.58	− 1.46 to 0.28	− 0.66	− 1.62 to 0.28	− 0.22	− 1.24 to 0.79	− 0.19	− 1.28 to 0.90

Others													
	Calcitonin												
	*P*^5^ = 0.25	*P*^6^ = 0.24		*P*^7^ = 0.25		*P*^6^ = 0.32		*P*^7^ = 0.33		*P*^6^ = 0.13		*P*^7^ = 0.15	
Pre-fasting	6.82 (37.9)	1		1		1		1		1		1	
Fasting	1.64 (1.09)	− 5.18	− 12.09 to 1.69	− 5.11	− 12.31 to 2.07	− 9.49	− 24.0 to 4.96	− 9.32	− 24.23 to 5.57	− 1.29	− 2.54 to − 0.04	− 1.32	− 2.65 to 0.02
Post-fasting	1.85 (1.71)	− 4.97	− 11.85 to 1.91	− 4.94	− 12.08 to 2.19	− 9.78	− 24.2 to 4.67	− 9.79	− 24.8 to 5.21	− 0.62	− 1.88 to 0.62	− 0.62	− 1.92 to 0.69

^1^Difference (between pre-fasting vs. fasting phases and pre-fasting vs. post-fasting phases) in percentage terms of biomarker concentration ≥ cut-off value or mean ± standard deviation

^2^Crude generalized least squares random-effects model or repeated measures logistic regression model to examine the association between biomarkers level with fasting or not state

^3^C.I. 0.95 confidence interval for Diff

^4^Adjusted generalized least squares random-effects model or repeated measures logistic regression model to examine the association between biomarkers level with fasting or not state

^5^p-values: null hypothesis of identical percentage differences of each biomarker concentration level for the pre-, per- and post-fasting or equality of means

^6^Crude p-value for association of biomarker plasma concentration ≥ cut-off value or for association difference of means for interval variables with co-factor

^7^Adjusted p-value for association of biomarker plasma concentration ≥ cut-off value or for association difference of means for interval variables with co-factor

#### Crude analysis

There was a significant shift in mean values across 80% of the biomarkers measured (19 of 25). Positive trends, which were absent prior to fasting, were discovered between haematological markers and a list of specific cytokines (GM-CSF, IL-1β, IL-1α, TGF-α and FIT-3L) during fasting. During fasting, the majority of biomarkers that were significantly altered by fasting (70%) fell below predetermined high cut off values. Among these biomarkers, the most significant changes were observed for TGF-α (− 53.8%) and MIP-1α (− 46.2%), respectively. Exceptions to the trends displayed by these biomarkers were the levels of IL-1β and IL-1α, as participants had biomarker concentrations above cut-off values of + 14.1% and + 48.7% for IL-1β and IL-1α respectively. During the post-fasting period, this was reduced to + 6.6% for the IL-1β but IL-1α remained at + 48.7%.

#### Regression (GLS and RMRL) models

Overall, plasma levels of biomarkers were altered by fasting and for some, corresponding trends persisted in the post-fasting period. During fasting, subjects exhibited elevated levels of haematological markers, and these associations persisted after adjustment. For platelet levels, fasting status was more strongly associated to increased numbers than post-fasting status (*p *< 0.0001). In addition, after adjustment, fasting promoted an increase in the plasma levels of GM-CSF (adjusted Coeff. = + 1.65, 95% CI [0.90–2.41]), IL-1α (adjusted Coef. = + 2.90, 95% CI [1.98–3.82]) and Flt-3L (adjusted Coef. = + 2.56, 95% CI [1.27–3.84]), and the trends for this molecules persisted in the post-fasting period. In contrast, the increased coefficient for WBC levels did not persist into the post-fasting period. In the adjusted analysis, fasting subjects tended to have higher IL-12p40 (adjusted Coef. = + 1.17, 95% CI [0.05–2.31]) concentrations. The contrary was observed for IFN-γ, IL-17, MIP-1α, ENA78 and fractalkine levels, as fasting had a buffering effect on their plasma concentrations. For TGF-α, MCP, eotaxin, MDC, GRO, CXC13, MIG and IP-10, a similar buffering effect was observed during fasting, which was preserved during the post-fasting period.

Overall, the shift occurring in biomarkers level appeared to be more important during the fasting period vs. post-fasting state (nearly all p values were below 0.05). This latter is particularly true for biomarkers that fluctuate during both fasting and post-fasting periods. Notably, IL-9 and IL-10 concentrations did not differ between fasting and baseline even after adjustment. In the adjusted analyses, our data reveals similar trends between both age categories for most of the biomarkers; discrepancies between both age groups were mainly observed in the post-fasting period. More specifically, changes in the plasma levels of TGF-α, sCD40L, APRIL, MIP-1α, MCP-2, MDC, GRO, eotaxin and IP-10 were more pronounced in the post-fasting period for younger adults than older participants.

### Older adults exhibit better resilience to fasting

In light of the differential resilience to RCF in older adults (Tables 3, 4) we furthered our exploration in this context. Aging studies would usually classify individuals as elderly when they reach 65 years old despite life expectancy is > 80 years. Since life expectancy in Pakistan is 66.5 years, we were more liberal in our classification of elderly subjects. We compared young individuals (< 35 years) with older adults in general (> 50 years) and alternatively further separated the older adults into elderly individuals (> 65 years).

**Fig. 4:**
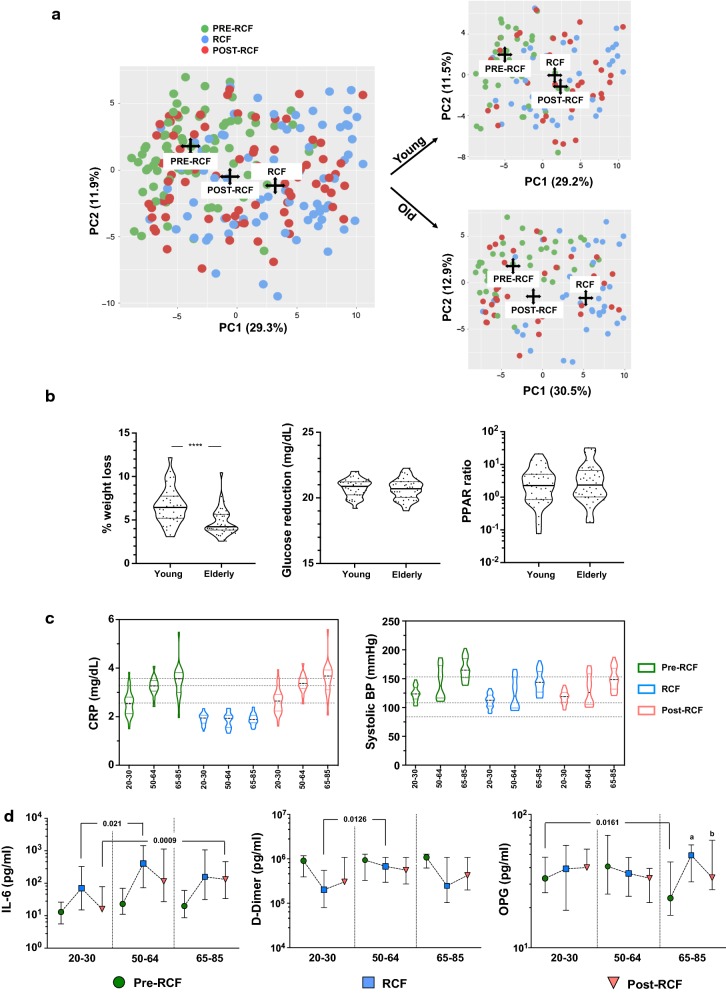
Age-dependent response to RCF. **a** Principal component analysis (PCA) of the biomarkers tested in the study for the three time points and similar analysis performed in young (n = 37, 26.5 ± 3.2 years, 20–30) and older participants (n = 41, 68.1 ± 8.6 years, 52–85). **b** Age-related adaptations to fasting and **c** the benefits of fasting on CRP and systolic BP were tested after stratification based on age [young (20–30), middle-aged (50–64) and old (65–85)]. **d** Similar analysis as in **c** showing IL-6, d-dimer and OPG. ^a^p = 0.005 significant difference with pre-fasting 65–85 (no difference with all groups together). ^b^p = 0.03 significant difference with pre-fasting 65–85 (no difference with all groups together)

We provide the results of the principal component analysis (PCA) analysis of biomarkers for all participants indistinctively of age (Fig. 4a). The principal component analysis (PCA) was applied so as to identify components reflecting young or old adults associated profile or signature. We observed an obvious shift in PC1 (29.3%) with fasting status, as depicted by the centroids of pre-fasting (green), fasting (blue) and post-fasting (red) data points from baseline. The top 20 molecules that contribute to each of the components are reported in Table 5. While PC1 consisted mainly of inflammatory and metabolic molecules, explanatory variables for PC2 are more closely related to the inflammasome. When both age groups were individually tested, young (n = 37, 26.5 ± 3.2 years, 20–30) and older adults (n = 41, 68.1 ± 8.6 years, 52–85), we observed a larger separation of biomarkers between post-fasting data points and baseline data points in younger adult, while data points for older adults in the post-fasting period clustered more closely with baseline values. These observations prompt us to conclude that older participants were more resilient to changes induced by fasting, i.e. that biomarkers from older adults equilibrate more rapidly than younger individuals in the post-fasting period. We also identified that weight loss (in percentage) was less significant in older individuals (Fig. 4b, p < 0.0001)—older adults lost 4.75 ± 1.52% (2.57–10.44) as compared to 6.62 ± 2.07% (3.09–10.44) for younger participants. Nevertheless, we did not observe any differential regulation for glucose or PPARβ. We further stratified the older adults into middle-aged (n = 18, 52–64 years) and elderly (n = 23, 65–85 years) as the oldest display higher levels and spread of biomarkers related to cardio-metabolic risks (Fig. 4c). As observed from the dotted lines within graphs, which indicate the median, levels of CRP and systolic BP display a stepwise increase with age. In our analysis of IL-6, d-dimer and OPG levels (Fig. 4d), which correspond to inflammatory, cardiovascular and metabolic domains respectively, we observed that IL-6 levels were more significantly up-regulated in the older age groups as compared to the youngest group. For d-dimer levels, the middle age group revealed the least pronounced reduction during fasting. Although OPG levels appeared unaltered by fasting in our global collective analysis (Fig. 2c, significant regulation was observed in the oldest group. Plasma OPG levels increased most significantly (27.3 ± 15.0 vs. 44.7 ± 23.4 pg/ml, p = 0.005) but post-fasting OPG levels were similar to the baseline of the youngest group (p = 0.4922, median: 33.1 vs. 33.6 pg/ml, respectively), showing a beneficial long-lasting effect.

**Table 5:** Parameters and their loadings of the principal components analysis

Old PC1		Old PC2		Young PC1		Young PC2	
Molecule	Loading	Molecule	Loading	Molecule	Loading	Molecule	Loading
GRO	− 0.397	IL1a	− 0.474	GRO	− 0.392	IL1RA	− 0.442
Leptin	− 0.349	GM.CSF	− 0.433	Leptin	− 0.355	IL1a	− 0.375
RANTES	− 0.339	IL1b	− 0.373	RANTES	− 0.345	GMCSF	− 0.321
Galectin	− 0.275	IL6	− 0.308	IP10	− 0.278	IL1b	− 0.302
MIP1a	− 0.247	GCSF	− 0.295	MDC	− 0.264	IL6	− 0.247
IP10	− 0.245	IL1RA	− 0.287	Galectin	− 0.222	MIP1b	− 0.237
MIP1b	− 0.233	MIP1b	− 0.156	MIP1a	− 0.206	GCSF	− 0.223
Fractalkine	− 0.224	TNFa	− 0.149	Fractalkine	− 0.203	Eotaxin	− 0.181
ENA78	− 0.199	IL10	− 0.149	MIP1b	− 0.194	ENA78	0.158
MDC	− 0.187	MIP1a	− 0.132	FGF	− 0.184	Galectin	− 0.154
CCL8	− 0.183	IL8	− 0.132	CCL8	− 0.168	Leptin	0.145
Eotaxin	− 0.152	MCP1	− 0.130	Eotaxin	− 0.160	FGF	− 0.141
FGF	− 0.149	IFNg	− 0.092	ENA78	− 0.152	IFNg	− 0.139
TGFa	− 0.148	IL5	− 0.085	MCP1	− 0.146	IL8	− 0.136
TNFa	− 0.135	Flt3L	− 0.083	sCD40L	− 0.135	sCD40L	− 0.131
MIG	− 0.114	FGF	− 0.081	TGFa	− 0.128	Flt.3L	− 0.130
IL10	− 0.106	Fractalkine	− 0.075	IL1a	0.120	PYY	− 0.130
IL1a	0.106	IGF1	− 0.065	MIG	− 0.114	IL17A	− 0.124
GMCSF	0.112	RANTES	0.063	IFNg	− 0.107	GRO	0.109
IL1RA	− 0.095	IP10	0.054	TNFa	− 0.102	RANTES	0.105

### Relationship between glycemia, CRP and nutrition during fasting

**Fig. 5:**
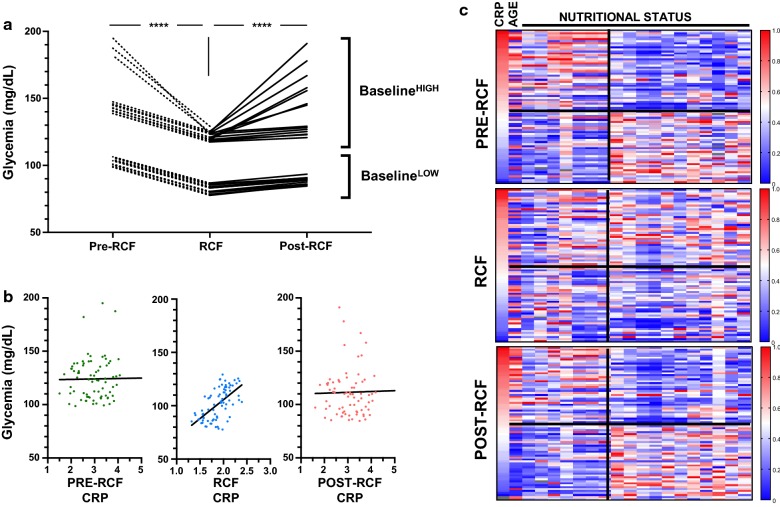
Relationship between inflammation and nutritional adaptation. **a** Glycemia regulation on fasting in individuals with highest vs. lowest glucose levels before the fasting period. **b** Relationship between glycemia and CRP levels. **c** Heatmap based on CRP as reference value and its relationship with selected biomarkers during the fasting study (age, B3 (mg), B6 (mg), PI index, NEAP, systolic BP, diastolic BP, BUN, energy (Kcal), protein (g), fat (g), saturated fats (g), calcium (mg), iron (mg), Phosph (mg), Vit D (μg), Vit B1 (mg), VIt B2 (mg), DII density)

Finally, we further characterised two important markers associated with inflammation (CRP) and metabolism (glucose) (Fig. 5). We separately plotted donors with the lowest and highest baseline fasting glucose levels (n = 15 each) over the three time-points and found that fasting was effective in reducing glucose levels in both participants with high (by 18.6 ± 7.6%) and low (by 19.6 ± 0.9%) baseline glucose levels. In the fasting period, glucose levels were still significantly higher (p < 0.0001) in the baseline^HIGH^ group (122.0 mg/ml ± 3.4; 117.6–129.2) as compared to the baseline^LOW^ group (82.8 mg/ml ± 2.9; 77.7–86.9). In contrast, while plasma CRP levels were significantly reduced in both CRP^high^ and CRP^low^ (4.17 ± 1.0 and 2.03 ± 0.21), we did not observe a significant difference in absolute CRP levels during fasting (1.85 ± 0.27 and 1.87 ± 0.21). This suggested a differential regulation of CRP and glucose levels in fasting versus non-fasting periods. When directly compared for each fasting period (Fig. 5b) we identified that both molecules were strongly positively correlated only during RCF (r = 0.6109, p < 0.0001). As changes in nutrient availability and sensing may affect the inflammatory profile, we examined the relationship between CRP levels and nutritional markers (heat map of correlation values, Fig. 5c). Nutritional data that display significant correlation with CRP levels at baseline (p < 0.05) was followed at the different stages of the study. As proof of concept, the data demonstrate how CRP levels are positively correlated with age in the pre-fasting phase, but this relationship is lost during fasting. Likewise, this trend was observed in the associations between CRP levels and the consumption of energy, protein, fat and most vitamins and minerals. Nevertheless, the relationships between CRP levels and phytochemical index (PI), net endogenous acid production (NEAP), and DII levels appeared to be stable during fasting. Altogether, this suggests interactions between physiological systems to be regulated by RCF and nutritional intake to differentially disturb these.

## Discussion

Recurrent circadian fasting is a traditional form of fasting annually practiced through the different stages of life. A large number of practitioners worldwide, estimated to 1 billion follow RCF. Since this process spans 29/30 continuous days, from sunset to dawn, it may be scientifically referred to as a form of recurrent circadian fasting. Although most fasting methods are meant to improve health through detoxification and regeneration, the few studies that exist on this topic highlight how RCF is often mismanaged and contributes to weight gain and inconclusive benefits [31]. We sought to dissect the key biological impact of RCF fasting in a cohort of Pakistani men, whom we know from past surveys to be compliant and maintain well-defined dietary practices.

### Statement of principal findings

Many physiological and psychological changes take place during fasting in response to changes in eating behaviour, frequency and sleeping pattern. Generally, the diet of participants in our study consisted of a combination of both healthy (high in fruits, vegetables, poultry, cruciferous or green leafy vegetables, tea, fruit juices, and whole grains), and processed (high in refined grains, red meat, butter, commercial meat products, high and low-fat dairy products, sweets, desserts, hydrogenated fats, soft drinks) food. In this cohort of fasting practitioners, we identified cardiometabolic benefits, differential regulation of inflammation, weight loss, changes in metabolic regulation (leptin, PPARβ) [32]. Moreover, in our characterisation of biological biomarkers (blood pressure, CRP, glucose levels), we observed that the elderly may receive more benefits from fasting than younger men, due either to the fact elderly are more susceptible to the extreme variations in nutriments intakes, to the longer experience and better management of fasting or simply to the fact they are the at-risk population [33]. Our study also describes the novel effect of fasting on biomarkers associated with physiological functions—such as orexin-A (wakefulness) [34], β-hydroxybutyrate (non-conventional metabolic pathways) [35] and PDGF-AA (aging/senescence) [36].

Specific to inflammation, the modulation of inflammatory markers (IL-6, TNFα, IL-8, CRP) is an important outcome of fasting as the current literature continues to support their association with morbidity and mortality. Pro-inflammatory molecules such as C-reactive protein (CRP), interleukin-6 (IL-6) and tumor-necrosis factor alpha (TNFα) are associated with major chronic conditions, including diabetes, cardiovascular diseases, and age-associated conditions such as frailty and dementia [37, 38]. Importantly, these molecules are differentially regulated during fasting, as CRP and TNFα were strongly downregulated but IL-6 and IL-8 reveal an inverse trend—dissociating the interactions between these signalling pathways could be important in discerning whether this is an effective homeostatic mechanism to maintain immune competency. Although the sustained or disproportionately high release of inflammatory molecules is often detrimental to cellular and tissue integrity [39], inflammation is a necessary tool that allows the immune system to facilitate the clearance of pathogen, restore tissue homeostasis and promote wound healing.

The post-fasting analysis of biomarker levels highlighted two trends; (i) biomarkers that return to baseline within a few weeks such as CRP and (ii) biomarkers that persist even up to a month, i.e. blood pressure, glucose level, d-dimer concentration, and inflammasome activity. Overall our study shows that RCF triggers a variety of beneficial health outcomes, if participants do not overcompensate (in terms of caloric intent and supplement balance) during the non-fasting period. This report supports observations from existing nutritional intervention studies (alternate day fasting, eTRF, caloric restriction, 5:2 diet, fat abstinence, Mediterranean diet), in demonstrating both short and long-term health benefits from fasting.

### Strengths and limitations of the study

This study benefits from access to a state-funded population survey and a high compliance rate, as recruitment was specifically in selected regions where nutritional intake during fasting periods was predictable and least divergent from their non-fasting periods [23, 24]. The latter behaviour is linked to multiple socioeconomic factors, including rurality, food availability, income and religiosity. Accordingly, none of the participants gained weight (from compensatory eating behaviour) and weight loss remained within an acceptable range during fasting. The low-income status of most participants, however, may be associated with a mild medical history, such as a higher frequency of both diagnosed undiagnosed cases of pre-diabetes/diabetes and/or hypertension. We also report significant numbers of participants with high systolic and diastolic blood pressures but who do not receive medication. A clear benefit of our study is the inclusion of a wide age-range, which allows us to stratify participants into multiple age groups: young (representing healthy non-at-risk individuals), middle-aged, and elderly (representing at-risk and potentially frail) individuals. Since we included biomarkers that spanned a diverse range of physiological systems, we were able to identify domains where fasting effects differed due to age [40, 41].

There are several caveats in our study, for example, only males were included in this study and our findings may not be extrapolated to women. This mainly results from difficulties in recruiting females concomitantly with males in a single study. In light of the beneficial effects of RCF at the cardiometabolic level and the higher prevalence of associated diseases in women, we have planned a dedicated study designed to investigate the beneficial impact of fasting on cardiometabolism, inflammation and hormonal regulation. Self-reporting on dietary intake may result into over- or underestimation of dietary intake. Nevertheless, we have restricted our study to a relatively small but sufficiently powered group of individuals (n = 78) to assess weight loss, our primary outcome. The number of participants also enabled to sustainably ensure compliance and supervision to facilitate accurate reporting and questionnaire administration. A subsequent study in other regions of the work with higher sample size would be useful in validating our main findings. Some biomarkers were not measured or did not reach sufficient quality, especially lipids and IGF-1/insulin and must be investigated in subsequent studies. A separate limitation is the absence of a separate non-fasting, age-matched, control population within the same study period. Because of the design of the study, it would be unethical to interfere with the fasting practices to obtain a comparable control group. The non-fasting individuals are usually those with chronic conditions (diabetes), acute infections or young adults, pregnant women and the frail elderly. Finally, we did not record changes in physical activity levels during the fasting period, which may be important due to associations between physical activity and the levels of investigated biomarkers, such as CRP and IL-6 [42].

### Comparison to other studies

Only a few recurrent fasting protocols have been investigated and the largest and longest report includes the monitoring of 1422 subjects who fasted for 4–21 days in accordance to the Buchinger fasting method. This extreme fasting method contributed to the loss of > 10 kg in males who fasted for more than 20 days. However, among 1422 subjects, only 37 (2.6%) were able to achieve this 20 days target. Moreover, we note that most metabolic changes did not persist for more than 15 days (e.g. glucose, triglycerides), possibly due to the extent of nutritional depletion. The dietary changes observed in our setting are more similar to other reports on RCF [43–45]. A decrease in daily calorie intake has been noted as one of the desired effects of RCF fasting [46]. Similar to what we observed, Salahuddi et al. [47] reported a significant drop in systolic and diastolic BP during fasting. However, conflicting reports suggested RCF fasting to have no effect on mean systolic and diastolic blood pressure in patients with hypertension, despite the reduced hydratation [48]. There is a lack of consensus on whether recurrent circadian fasting improves [47] or worsens lipid profiles [49] probably due to health heterogeneity in the studied populations [17]. The same applies for HDL [19, 50]. Adlouni et al. [51] conducted a study where a significant reduction in total cholesterol and triglyceride levels was seen during RCF fasting, but suggested that these effects may be specific to or could be explained by the Mediterranean dietary habits in Morocco.

Several studies on intermittent fasting have revealed a positive effect on inflammatory state and relieves the inflammatory burden in healthy individuals and in the contexts of pregnancy, cardiovascular diseases, asthma, brain disorders and as part of intense physical training [52]. However, only a few studies demonstrated reduced IL-6, and TNF-α levels during RCF (p < 0.05) as most studies only focused on CRP [53, 54]. The role of the inflammasome in the RCF-induced regulation of inflammation is still to be proven. While IL-1β is up-regulated following RCF this does not preclude that overall inflammation is reduced, due to the up-regulation of anti-inflammatory molecules such as IL-10, TGF-β, and receptors to IL-6, TNF-α, IL-18 among others. For instance, we show IL-6 up-regulation which aligns with IL-1β up-regulation and platelets elevation. Studies have shown the release of IL-6R by platelets [55] and their ability to mediate inflammatory signalling via IL-1β synthesis [56]. The emerging role of platelets in controlling inflammation [57] and related cardiometabolic diseases [58, 59] deserves more focus in the context of fasting [60]. Experimental studies by Sack et al. suggested that timing of measurement is extremely important to study inflammasome activation [61, 62]. It is likely that the timing for blood collection and the fasting/feeding period difference between studies accounts for discrepancies. Taken together, these results support the perception of reduced inflammation during RCF fasting. In addition to the modulation of these molecules, we also observed a significant downregulation in IP-10 and PDGF-AA after fasting; to the best of our knowledge, no previous study on fasting has evaluated the impact of fasting on these two chemokines. IP-10, initially identified as an IFN-γ-inducible chemokine and functionally categorized as a pro-inflammatory chemokine, is known to regulate immune responses by activating and recruiting leukocytes including T cells, eosinophils, monocytes and NK cells [63]. Moreover, IP-10 is secreted by a variety of cells including monocytes, neutrophils, endothelial cells, keratinocytes, mesenchymal cells, dendritic cells, astrocytes and hepatocytes. Other research has revealed that intrahepatic and circulating IP-10 is associated with obesity and insulin resistance in patients with chronic hepatitis C virus (HCV) infection and in patients with HCV/human immunodeficiency virus (HIV) co-infection [64]. A major issue in such biomarker analysis is the identification of the source of inflammation. In the context of fasting, we envision immune cells and organs to adapt differently at the inflammatory level. Including exosome-derived inflammatory measures would enable to identify the origin of the circulating biomarkers [65, 66] and better understand the impact of fasting on the several physiological systems.

### Possible explanations of findings

Recurrent circadian fasting may alter the circadian consumption and distribution of energy and other nutrients for the fulfilment of daily requirements. For example, breakfast accounts for up to 30% of the daily calorie intake and the post-sunset meal constitutes 60% of the daily energy intake [13] but larger datasets should be gathered on the physiological consequences [14]. A recent meta-analysis suggested that breakfast may not be recommended for individuals whose intention is to lose weight [15]. On a related note, recent studies have demonstrated a negative impact of forgoing breakfast [16], and late night eating habits on cardiometabolic health [18]. Alternatively, the American Heart Association recommends that dietary habits which pay mindful attention to the factors of timing and frequency (which also implies quantity and quality) promote the better management of cardiometabolic risks. As RCF adheres to a strict schedule, the beneficial effects observed in our study supports the latter conclusion.

A main concern of RCF fasting studies relates to the radical dietary readjustments that occur during this period, particularly concerning carbohydrate and protein intake [44]. This may be due to higher consumption of dates, honey, sweets and soft drinks as often noted in middle-eastern countries. The rapid elevation of socio-economic status and mal-adaptation to western diets also contribute to a lower compliance to nutritional intake recommendations during fasting. Differences in physical activity levels may also account for some of the discrepancies in observations between studies. While some societies reveal diminished physical activity levels during RCF, some groups of individuals, especially the elderly, participate in longer periods of moderate physical activity due to their active participation in mindfulness-related activities that centre on prayer [67]. Related to the latter, sleeping patterns may also be interrupted by fasting, as suggested by the heterogeneity of orexin-A levels in our study. Nevertheless, RCF compliance with recommended lifestyle (maintaining daily activities and unchanged/reduced carbohydrate and protein intake) in our study, may lead to plausible health benefits that relate to weight loss and inter-systems regulation. The extent of RCF-induced benefits may differ in other studies due to factors associated with geographical and cultural predisposition (Middle-East, Eurasian, South-East Asia, immigrants in western countries). It has been proposed that health outcomes resulting from RCF are affected by various factors, including variations in the length of fasting (e.g. 16–20 h in the same year), daytime temperature [31, 68], dietary habits/customs during RCF, fluid consumption (e.g. sweetened drinks or water/juices), sleep duration and pattern [69, 70], working hours, job conditions, physical activity levels [71] and ritualistic as well as social behaviours [72].

A possible mechanism for the lowering of inflammation and oxidative stress markers with RCF may relate to reduced activation of the insulin/insulin-like growth factor 1 (IGF-1) axis, which predisposes individuals—particularly the obese—to inflammatory and oxidative stress [73]. Behaviours associated with obesity may also sustain chronic diseases through the induction of pro-inflammatory cytokines and insulin and insulin/IGF-1 resistance—both processes further contributes to the increased production of reactive oxygen species and residual inflammation. Although we could not measure insulin resistance and IGF-1 levels in our study, other studies have associated RCF with improved insulin levels and sensitivity in obese individuals with metabolic syndrome [74]; the sustained reduction in glucose levels observed in our study may act as supporting mechanism for the latter findings.

Wegman and colleagues [75] reported an increase in the expression of SIRT3, which encodes for NAD-dependent deacetylase Sirtuin-3 (reduced during aging) during fasting, which aligns with our observed down-regulation of PDGF-AA (associated with SASP). The significant reduction of total body and visceral fat reported at the end of RCF in our study and others [76], may further lead to adipocyte dormancy and reduced secretion of adipokines [77]. During RCF, drastic changes in the timing and frequency of fluid and food intake were also observed in our study (Fig. 1a)—this contributes to chronobiological changes in the circadian distribution of body temperature, melatonin, cortisol, blood glucose levels, as well as day- and night-time wakefulness [68]. These physiological changes have been reported to benefit several systems and reduce overall physiological stress [78].

## Conclusion

Recurrent circadian fasting exists as a unique model to explore the impact of annual recurrent food restriction (several weeks) within a fixed duration (similar to eTRF) on multiple physiological systems [79]. RCF is a safe lifestyle modification involving specific dietary and lifestyle modifications, such as changes in the frequency, quantity and quality of food intake, nocturnal food consumption, and sleep cycle [80]. Nevertheless, different studies have reported contrasting impacts of RCF on health [74, 80]. Overall, the impact of RCF provides short-term protection against low-grade systemic inflammation and oxidative stress. This reduction in inflammatory and oxidative stress markers could be attributed to weight loss during the fasting month [81]. Furthermore, increased body weight is associated with increased levels of inflammation and oxidative stress in the human body, with elevated IL-6, IL-1, and TNF-α among the common trends that predisposes obese individuals to metabolic malmanagement [46, 82] and that can be impacted by conditions such as diabetes [83, 84]. Data on the dietary behaviour of fasting individuals in RCF are scarce and tend to be focused on the cataloguing of food items or nutrients. From the perspective of public health, the collection of such data are crucial to setting appropriate clinical guidelines to harness the benefits of RCF [85]. Since there is no consensus on optimal dietary behaviour during the Ramadan fasting period, particularly from a health perspective, future studies should focus on developing these recommendations based on observed improvements in biomarkers during the fasting period. This endeavour is important as the target population is large (~ 1 billion) and encompasses individuals who are acquiesced to or agreeable with fasting. However, future studies should focus on understanding the benefit of RCF in specific populations stratified by age and health conditions.

## Additional files

**Additional file 1.** Additional Tables S1, S2.

**Additional file 2: Figure S1.** Heat map of clinical and nutritional readouts of the cohort before, during and after the fasting period. **Figure S2.** A) Serum levels of G-CSF, Galectin, sCD14 measured by Multiplex. B) Creatinine and BUN in plasma were measured by clinical laboratory tests.

## Data Availability

The data generated and analysed for this study can be made available by the corresponding author on reasonable request.

## Abbreviations

ALAT: l-alanine aminotransferase
APRIL: a proliferation-inducing ligand
ASAT: l-aspartate aminotransferase
BMI: body mass index
BUN: blood urea nitrogen
CRP: C-reactive protein
ELISA: Enzyme-Linked Immunosorbent Assay
FFQ: Food Frequency Questionnaire
GM-CSF: granulocyte-macrophage colony-stimulating factor
GRO: growth-regulated oncogene
Hb: haemoglobin concentration
HCT: haematocrit
HGF: hepatocyte growth factor
IF: intermittent fasting
IL: interleukin
KPK: Khyber PakhtunKhwa
LDL: low-density lipoproteins
LOD: limit of detection
MCP-1: Monocyte Chemoattractant Protein-1
MCV: mean corpuscular volume
MDC: Macrophage-Derived Chemokine
MIG: monokine induced by gamma
MIP: macrophage inflammatory proteins
MPV: mean platelet volume
NEAT: nutrition, education, awareness and training
OPG: osteoprotegerin
PCA: principal component analysis
PDGF-AA: platelet-derived growth factor AA
PPAR: peroxisome proliferator-activated receptors
PPARs: peroxisome proliferator-activated receptors
RCF: recurrent circadian fasting
sCD: serum cluster of defferintiation
TGF: transforming growth factor
TNFa: tumor necrosis factor-a
TRF: time-restricted feeding
WBC: white blood cells
