# Supplementary material for: Recurrent circadian fasting (RCF) improves blood pressure, biomarkers of cardiometabolic risk and regulates inflammation in men
Source: J Transl Med. 2019 Aug 19;17:272. doi: 10.1186/s12967-019-2007-z (PMC6700786; doi:10.1186/s12967-019-2007-z)
Supplement: Supplementary file 1 — Additional file 1. Additional Tables S1, S2. [file 12967_2019_2007_MOESM1_ESM.docx]

**Table S1**

| **FOODS/BEVERAGES** | **DESCRIPTION** | **Mean amount consumed pre-Fasting** | **Mean amount consumed during Fasting** |
| --- | --- | --- | --- |
| Wheat Bread | wheat flour (whole), salt | 204.6 g | 234.8 g |
| Wheat Paratha | Wheat flour (whole), salt, oil or ghee (hydrogentaed fat) | 120.9 g | 145.7 g |
| Beef/Mutton Curry | Beef/Mutton, tomatoes, onion, garlic, oil or ghee, salt, spices, | 145.5 g | 180.9 g |
| Beef plus potato Curry | Beef, tomatoes, onion, potatoes, salt, spices, | 102.9 g | 107.4 g |
| Beef plus some vegetable Curry | Beef, tomatoes, onion, garlic, oil or ghee, salt, spices, | 43.5 g | 35.7 g |
| Vegetable curry | vegetables (veg. okra, pea, lentils, green leafy veg), onion, garlic, oil or ghee, salt, spices, | 101.8 g | 23.6 g |
| Dal Curry (Dal=pulses, gram, lentils etc) | pulses, onion, garlic, oil or ghee, salt, spices, | 65.8 g | 21.6 g |
| Pakora | flour of dried pulses, onion, garlic, oil or ghee, salt, spices, | 21.6 g | 69.5 g |
| samosa | wheat flour, potatoes, onions, tomatoes, spices, oil or ghee | 13.9 g | 78.4 g |
| fruit chaat | various fruits pieces mixed with yogurt | 4.5 g | 72.3 g |
| Chatni (condiment) | tomatoes, green pepper, garlic, salt (ground together) and lemon juice is mixed | 21.2 g | 28.7 g |
| Pickles |  | 2.1 g | 43.2 g |
| Kajoor (dates) |  | 1.0 g | 65.3 g |
| Sugar sharbat | sugar, water, ice, added with some synthetic juice (e.g. Rooh Afaz, Jam e Shereen etc) | 54.6 g | 276.8 g |
| Lasi (laban), Milk water | yogurt, water, ice | 45.9 g | 254.7 g |
| Gur Sharbat (Gur is brown sugar, molasse) |  | 67.8 g | 345.6 g |
| Tea with Milk |  | 209.6 g | 389.6 g |
| Baryani | Rice, oil or ghee, spices, | 32.1 g | 27.5 g |
| Boiled Rice without spices (Particularly for sahoor) |  | 6.5 g | 78.9 g |
| musk-melon, mangoes, banana, apples, grapes |  | 18.6 g | 76.5 g |
| **SOME UNIQUE FOODS/FUNCTIONAL FOODS** |  |  |  |
| sat poodina | peppermint extract | rare | common |
| sat ajwain | Carom Seeds extract | rare | common |
| sat lamboo | lemon extract | rare | common |
| Lemon |  | rare | common |
| gonde kateera | Tragacanth gum | rare | common |
| sat aspaghol | ispaghula husk | rare | common |

**Table S2. Mean (s.d.) intake of Minerals and Vitamins at three time points.**

| **Parameters** | **Pre-Fasting** | **Fasting** | **Post-Fasting** | **P-value** |
| --- | --- | --- | --- | --- |
| **Minerals** | | | | |
| Calcium (mg/d) |  |  |  |  |
| *Mean (s.d.)* | 413.7 (149.5) | 368.6 (109.5) | 369.6 (160.4) | <0.0001 |
| Zinc (mg/d) |  |  |  |  |
| *Mean (s.d.)* | 8.4 (4.5) | 11.4 (3.4) | 8.4 (2.1) | 0.38 |
| Magnesium (ug/d) |  |  |  |  |
| *Mean (s.d.)* | 194.3 (42.1) | 205.1 (45.4) | 207.3 (45.1) | 0.34 |
| Phosphorus (mg/d) |  |  |  |  |
| *Mean (s.d.)* | 649.3 (193.1) | 668.0 (121.3) | 655.2 (195.4) | <0.0001 |
| Selenium (ug/d) |  |  |  |  |
| *Mean (s.d.)* | 16.2 (6.9) | 17.5 (7.2) | 16.8 (7.8) | 0.0001 |
| Copper (ug/d) |  |  |  |  |
| *Mean (s.d.)* | 452.8 (126.5) | 447.9 (126.3) | 452.8 (102.9) | 0.0011 |
| Manganese (ug/d) |  |  |  |  |
| *Mean (s.d.)* | 1.7 (0.3) | 1.8 (0.09) | 1.5 (0.5) | <0.0001 |
| **Parameters** | **Pre-Fasting** | **Fasting** | **Post-Fasting** | **P-value** |
| **Vitamins** | | | | |
| Vitamin A (ug/d) |  |  |  |  |
| *Mean (s.d.)* | 212.4 (76.7) | 221.2 (78.2) | 199.1 (56.7) | <0.0001 |
| Vitamin C (mg/d) |  |  |  |  |
| *Mean (s.d.)* | 30.5 (9.3) | 24.1 (11.4) | 23.1 (4.8) | <0.0001 |
| Vitamin B1 (mg/d) |  |  |  |  |
| *Mean (s.d.)* | 0.8 (0.3) | 1.0 (0.3) | 0.8 (0.2) | <0.0001 |
| Vitamin B2 (mg/d) |  |  |  |  |
| *Mean (s.d.)* | 0.6 (0.3) | 1.0 (0.6) | 0.7 (0.3) | <0.0001 |
| Vitamin B3 (mg/d) |  |  |  |  |
| *Mean (s.d.)* | 18.8 (10.0) | 19.5 (9.1) | 19.1 (10.2) | <0.0001 |
| Vitamin B6 (mg/d) |  |  |  |  |
| *Mean (s.d.)* | 1.4 (1.0) | 1.7 (1.1) | 1.5 (1.0) | <0.0001 |
| Biotin (ug/d) |  |  |  |  |
| *Mean (s.d.)* | 19.3 (2.9) | 20.0 (3.1) | 17.9 (3.1) | <0.0001 |
| Folate (ug/d) |  |  |  |  |
| *Mean (s.d.)* | 322.5 (20.2) | 337.3 (22.4) | 303.7 (22.1) | <0.0001 |
| Cholin (mg/d) |  |  |  |  |
| *Mean (s.d.)* | 377.5 (23.4) | 340.4 (62.3) | 377.2 (27.1) | <0.0001 |
| Vitamin E (mg/d) |  |  |  |  |
| *Mean (s.d.)* | 11.0 (5.1) | 10.7 (5.4) | 11.0 (5.2) | 0.22 |
| Vitamin K (ug/d) |  |  |  |  |
| *Mean (s.d.)* | 64.8 (1.6) | 64.0 (2.3) | 64.4 (2.) | <0.0001 |
