# Supplementary material for: Recurrent circadian fasting (RCF) improves blood pressure, biomarkers of cardiometabolic risk and regulates inflammation in men
Source: J Transl Med. 2019 Aug 19;17:272. doi: 10.1186/s12967-019-2007-z (PMC6700786; doi:10.1186/s12967-019-2007-z)
Supplement: Supplementary file 2 — Additional file 2: Figure S1. Heat map of clinical and nutritional readouts of the cohort before, during and after the fasting period. Figure S2. A) Serum levels of G-CSF, Galectin, sCD14 measured by Multiplex. B) Creatinine and BUN in plasma were measured by clinical laboratory tests. [file 12967_2019_2007_MOESM2_ESM.pptx]

## Slide 1
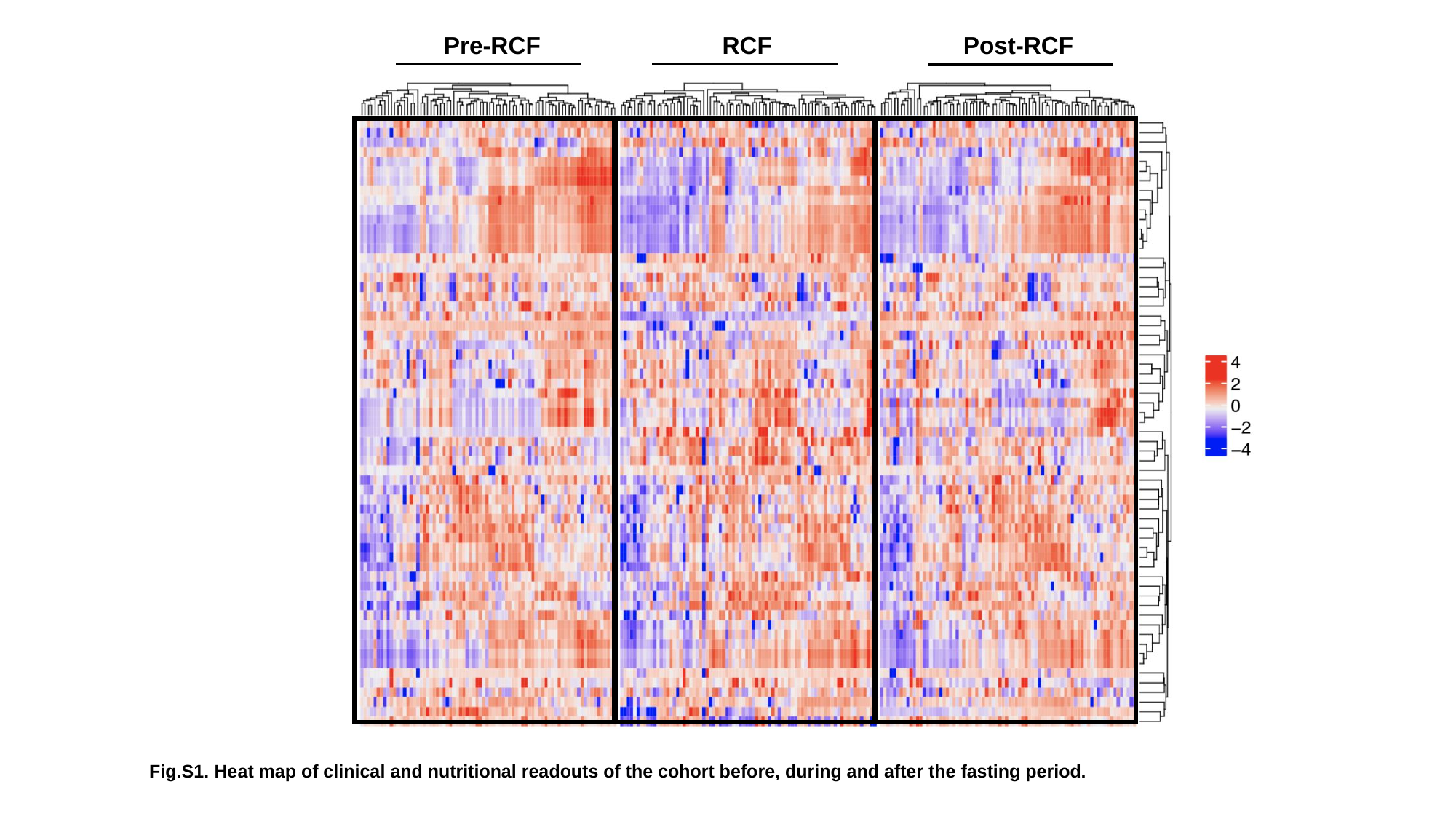

RCF
Post-RCF
Pre-RCF
Fig.S1. Heat map of clinical and nutritional readouts of the cohort before, during and after the fasting period.

## Slide 2
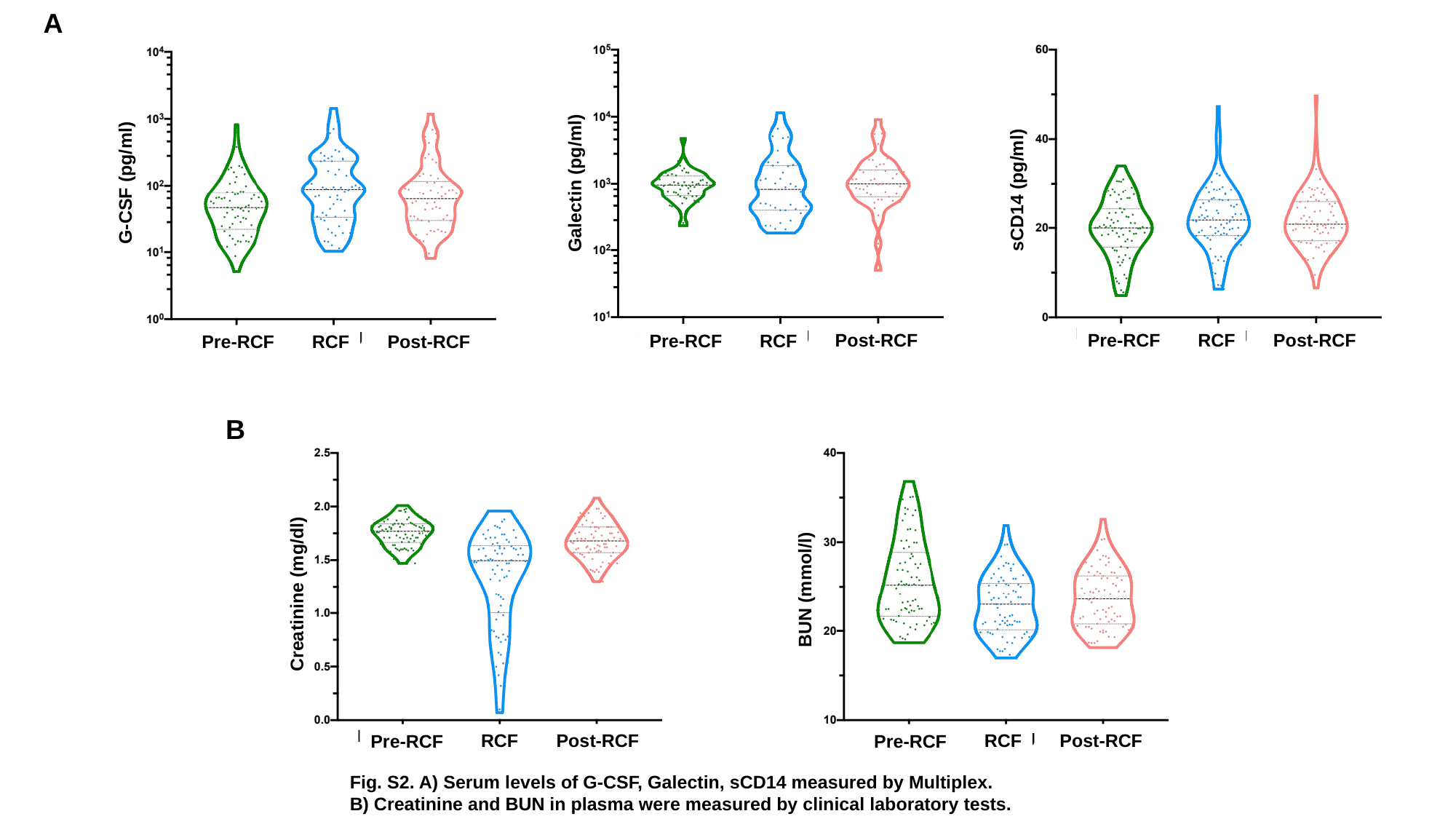

A
G-CSF (pg/ml)
Galectin (pg/ml)
sCD14 (pg/ml)
Post-RCF
RCF
Pre-RCF
Post-RCF
RCF
Pre-RCF
Post-RCF
RCF
Pre-RCF
B
BUN (mmol/l)
Creatinine (mg/dl)
Post-RCF
Post-RCF
RCF
RCF
Pre-RCF
Pre-RCF
Fig. S2. A) Serum levels of G-CSF, Galectin, sCD14 measured by Multiplex.
B) Creatinine and BUN in plasma were measured by clinical laboratory tests.
